# Anti-Inflammatory and Anti-Arthritic Efficacies of an Indian Traditional Herbo-Mineral Medicine “Divya Amvatari Ras” in Collagen Antibody-Induced Arthritis (CAIA) Mouse Model Through Modulation of IL-6/IL-1β/TNF-α/NFκB Signaling

**DOI:** 10.3389/fphar.2019.00659

**Published:** 2019-07-01

**Authors:** Acharya Balkrishna, Sachin Shridhar Sakat, Kheemraj Joshi, Sandeep Paudel, Deepika Joshi, Kamal Joshi, Ravikant Ranjan, Abhishek Gupta, Kunal Bhattacharya, Anurag Varshney

**Affiliations:** ^1^Drug Discovery and Development Division, Patanjali Research Institute, Haridwar, India; ^2^Center for Nanotechnology and Nanotoxicology, Harvard T.H. Chan School of Public Health, Boston, MA, United States; ^3^University of Patanjali, Patanjali Yog Peeth, Haridwar, India

**Keywords:** rheumatoid arthritis, inflammation, *in vivo* studies, cytokines, herbo-mineral formulation, Ayurveda, Divya Amvatari Ras

## Abstract

Rheumatoid arthritis (RA) is defined as a chronic autoimmune inflammatory disorder that causes damage to limb joints and progressive injuries to secondary organs. Medical practitioners prescribe Methotrexate (MTX) as standard care medicine for treating RA. However, the long-term application of MTX has shown to have adverse health-related effects. Divya Amvatari Ras (DAR), an Indian Ayurvedic herbo-mineral formulation, has been described in ancient texts to provide relief from RA inflammation associated distress. Therefore, in the present study, we explored the biocompatibility, anti-inflammatory, and anti-arthritic efficacy of DAR using *in vivo* and *in vitro* disease models. Using carrageenan (CA)-stimulated Wistar rat paw edema model, we showed a reduction in inflammation-induced paw edema at human equivalent dose of DAR. Anti-rheumatic efficacy of DAR was studied using collagen-antibody cocktail (C-Ab) Induced Arthritis (CAIA) mouse model. The onset of RA in the CAIA mice was determined using parameters such as the increase in arthritis score, and induction of disease associated lesions in the ankle and knee joints, and increase in mechanical and thermal hyperalgesia. Treatment of CAIA animals with a human equivalent dose of DAR significantly reversed the RA-associated pathogenesis. These effects were comparable with the standard of care RA drug, MTX. DAR acted at multiple levels of inflammation associated with RA to reduce progressive pathogenesis. Animal serum biochemistry showed DAR was capable of ameliorating RA induced increase in liver enzyme Alanine Aminotransferase (ALT) and pro-inflammatory cytokine interleukin 6 (IL-6). In the lipopolysaccharide stimulated THP-1 cells, DAR was found to inhibit the release of IL-6, IL-1β, TNF-α, and upstream inflammatory gene regulatory protein, NFκB. The study endorsed the anti-arthritic and anti-inflammatory activity of the Indian Traditional herbo-mineral medicine, DAR. These results also confirm that DAR was highly biocompatible and would show minimal health-related side effects than those associated with standard of care MTX. Taken together, we show that the DAR could be utilized as a promising alternative or complementary therapy for treating rheumatoid arthritis.

## Introduction

Rheumatoid arthritis (RA) is a systemic autoimmune disease that causes chronic inflammation in the limb joints and other secondary organs. While it is more prevalent in the female population, intrinsic and extrinsic factors play a key role in the development of RA (Smolen et al., 2018). Prolongation of RA is associated with pathogenesis such as cartilage damages and bone erosions (Smolen et al., 1995). Under chronic and untreated conditions, RA can lead to severe and irreversible damage to the joints leading to permanent disabilities.

Site-specific pathogenesis of RA disease is centered around the role of localized systemic factors that affect particular anatomical sites, along with localized mechanical elements (Ospelt and Frank-Bertoncelj, 2017). Soluble mediators such as pro-inflammatory cytokines, chemokines, leukotrienes, prostaglandins, citrullinated proteins, and collagen-degrading proteases like matrix metalloproteinase act as precursors in inducing RA pathogenesis in the synovial region (McInnes and Schett, 2007; Brennan and McInnes, 2008; Bartok and Firestein, 2010; McInnes and Schett, 2011; Apel et al., 2018; Smolen et al., 2018). These mediators are released from the fibroblast-like synoviocytes and immune cells such as resident macrophages, monocytes, and neutrophils. Currently, there are no long-term relief treatments available for the controlling RA associated pathogenesis. Topical and oral application of corticosteroids, non-steroidal anti-inflammatory drugs (NSAIDs), disease modifying anti-rheumatic drugs (DMARDs), and cell signaling inhibitors may cause temporary relief. However, their prolonged application may have severe health-related side effects. One of the most commonly employed DMARD is Methotrexate (MTX), an antifolate drug. MTX has a 1000-fold affinity to dihydrofolate reductase compared to folate and inhibits the conversion of dihydrofolate to tetrahydrofolate. Inhibition of tetrahydrofolate synthesis by MTX leads to cessation of cell division and other protein synthesis. Besides acting as an anti-inflammatory agent, MTX also acts as anti-cancer drug and has been listed as an essential medicine by the World Health Organization (Howard et al., 2016). Clinically, MTX is prescribed in low doses of 10–25 mg/week (Weinblatt, 2013). However, a few clinical studies have reported a low-dose toxicity of MTX in elderly patients and patients with slow metabolic clearance. These observed adverse effects have been attributed to the bioaccumulation of MTX and its metabolites in tissues (Shaikh et al., 2018; Arakawa et al., 2019).

Amvatari Ras is a traditional Indian herbo-mineral medicine that has been cited for treating *Amvata* (Sanskrit word for RA) in several ancient Indian Ayurveda texts [Rasendra Chintamani (Classical Text), 15th century A.D.; Bhaishajya Ratnawali (Classical Text), 18th century A.D.] and The Ayurvedic Formulary of India 2003 (Ministry of Health and Family Welfare, Government of India, 2003) for the treatment of *Amvata*. This herbo-mineral formulation contains herbal extracts of *Terminalia chebula, Terminalia bellirica, Emblica officinalis, Plumbago zeylanica, Commiphora mukul*, and *Ricinus communis*, along with herbally processed mercury (Hg) and sulfur (S) (**Table 1**). Clinical studies earlier using Amvatari Ras (Bhinde et al., 2016) have shown a marked reduction in the inflammatory response and relief from RA-associated symptoms in human subjects.

**Table 1:** Weight percentage of components in the herbo-mineral formulation, Divya Amvatari Ras (DAR), as per classically described manufacturing process.

DAR components	Scientific names	Weight (%)
Harad badi	*Terminalia chebula*	6.50
Behara chilka	*Terminalia bellirica*	6.50
Amla sukha	*Emblica officinalis*	6.50
Chitrak mool	*Plumbago zeylanica*	19.51
Shodhit Parad	Herbal processed mercury	4.88
Shodhit Gandhak	Herbal processed sulfur	9.75
Shuddh Guggul	*Commiphora mukul*	24.34
Arand Oil	*Ricinus communis*	7.80
Babool gond	*Vachellia nilotica*	1.56
Other inert components		Q.S.

CA-stimulated paw edema is a well-established animal model for studying inflammation. Sub-plantar injection of CA in animals causes a consistent bi-phasic edema in 7- to 8-week-old animals. These responses are largely based on the stimulation of cyclooxygenase-2 (COX-2) and nitric oxide synthase (iNOS) (Posadas et al., 2004). CA-induced inflammation animal models have been used extensively for studying the anti-inflammatory behavior of pharmaceutical and natural formulations. Collagen type II is the major component of the joint cartilage matrix proteins. In collagen-antibody-induced arthritis (CAIA) animal models, systemic administration of a cocktail of collagen monoclonal antibodies (C-Ab) targets the various regions of collagen type II (Khachigian, 2006). CAIA animals are further stimulated by lipopolysaccharide (LPS) for induction of joint inflammation and development of RA associated pathogenic features (Staines and Wooley, 1994). CAIA animal model has been extensively used for the testing of anti-inflammatory and anti-arthritic drugs and formulations.

In the present investigation, Divya Amvatari Ras (DAR) was studied for its anti-inflammatory and anti-arthritic potential using CA stimulated Wistar rat paw edema model and C-Ab and LPS stimulated arthritis Balb/c mouse model. In the CA-stimulated rats, the anti-inflammatory potential of the DAR was tested for changes in paw edema. In the C-Ab and LPS-stimulated mice, changes in RA-associated clinical pathogenicities such as arthritis score, paw thickness, knee-joint thickness, and mechanical and thermal hyperalgesia were studied along with histopathological and radiological screening. MTX was used as a positive control to verify the experimental model; however, comparison of single compound reference drug with nature sourced formulation is not the aim of this study. The study showcases the effectiveness of DAR as an anti-arthritic medication with no health-associated side effects.

## Materials and Methods

### Chemicals and Reagents

Classically Amvatari Ras is manufactured by Divya Pharmacy, Haridwar, India, under the brand name “Divya Amvatari Ras” (DAR). For the presented studies, DAR (Batch no: A-AVR008, manufacturing date: Oct 2017) was sourced from the Divya Pharmacy. Based on the manufacturing protocol, the weight percentage of different components of DAR is mentioned in **Table 1**. High Performance Liquid Chromatography (HPLC)-based phytochemical analysis of DAR is shown in **Supplementary Figure 1** (analytical method provided as Supplementary Information). The manufacturer undertook regular quality checks for the purity of input components, as per their standard operating procedure. 5-Clone Cocktail antibodies (cat no-53040) and LPS (*Escherichia coli* strain 0111: B4; cat no-9028) were purchased from Chondrex, Inc. WA, USA. λ-Carrageenan, indomethacin, and MTX were procured from Sigma Aldrich, St. Louis, MO, USA. Hematoxylin, potassium aluminum sulfate dodecahydrate, and mercury (II) oxide red were purchased from Merck India Pvt Ltd, Mumbai, India. Safranin and Fast green were procured from Loba Chemie Pvt. Ltd, Mumbai, India. Eosin Yellow and Ferric chloride were purchased from HiMedia Laboratories, Mumbai, India. All other chemicals and reagents used in the study were of the highest commercial grade. For cell culture work, RPMI-1640 cell culture media, fetal bovine serum (FBS), antibiotics, and other reagents were purchased from Thermo Fisher Scientific, India.

### Experimental Animals

Male Balb/c mice (20–30 g) were procured from Charles River Laboratory licensed supplier, Hylasco Biotechnology Pvt. Ltd, Hyderabad, India. Male Wistar rats (160–180 g) were obtained from Liveon Biolabs Pvt. Ltd, Bangalore, India. All the animals were housed in polypropylene cages in controlled room temperature 22 ± 1°C and relative humidity of 60–70% with 12 h:12 h light and dark cycles in a registered animal house (Registration number: 1964/PO/RC/S/17/CPCSEA). The animals were supplied with standard pellet diet (Purina Lab Diet, St. Louis, MO, USA) and sterile filtered water *ad libitum*. The study protocol was approved by the Institutional Animal Ethical Committee of Patanjali Research Institute vide IAEC approval numbers PRIAS/LAF/IAEC-032 and PRIAS/LAF/IAEC-009. All the experiments were performed using relevant guidelines and regulations.

### Dose Calculation for *In Vivo* Experiments

The animal equivalent doses of DAR for rat and mouse studies were estimated based on the body surface area of the animals. The human recommended dose of the DAR is two tablets twice a day. The average weight of each tablet is 550 mg. Accordingly, total human dose is 2,200 mg/60 kg/day (36.66 mg/kg/day). Animal equivalent doses (mg/kg) for rat and mouse were calculated by multiplying human equivalent dose (mg/kg) by factors 6.2 and 12.3, respectively (Nair and Jacob, 2016). Resultant therapeutic equivalent doses (TEDs) for rat and mouse were found to be 227 and 454 mg/kg, respectively. Therefore, 200 mg/kg (round off) and 454 mg/kg have been taken as rat and mouse doses (human equivalent dose), respectively, for pharmacological studies.

### Assessment of Anti-Inflammatory Activity Using CA-Induced Rat Paw Edema Model

CA-induced rat paw edema model was tested according to the modified methods described earlier (Sakat et al., 2014). Male Wistar rats were divided into different groups of eight animals, based on basal paw volume (0 h) measured using Plethysmometer 37140 (Ugo Basile, Italy). Inflammation was induced by the subcutaneous injection of λ-CA (0.1 ml of 1% solution in normal saline) into the plantar side of the left hind paw. The paw was marked with ink at the level of lateral malleolus and volume was measured up to the mark at 1, 2, 3, 4, and 5 h after injection of CA for all the animals (Sakat et al., 2014). Animals were treated orally with vehicle or DAR (at 200 mg/kg) or indomethacin (at 10 mg/kg), 1 h before CA challenge. Paw edema was calculated by subtracting 0 h (basal) paw volume from the respective paw volumes at 1, 2, 3, 4, and 5 h. The change in inﬂammation (%) was calculated for each animal using the following formula: [(paw volume of respective time point (ml) − paw volume of zero-time point (ml))/paw volume of zero-time point (ml)] × 100 (Sakat et al., 2014).

### Assessment of Anti-Arthritic Potential

#### Induction of Arthritis in Balb/c Mice

**Figure 1:**
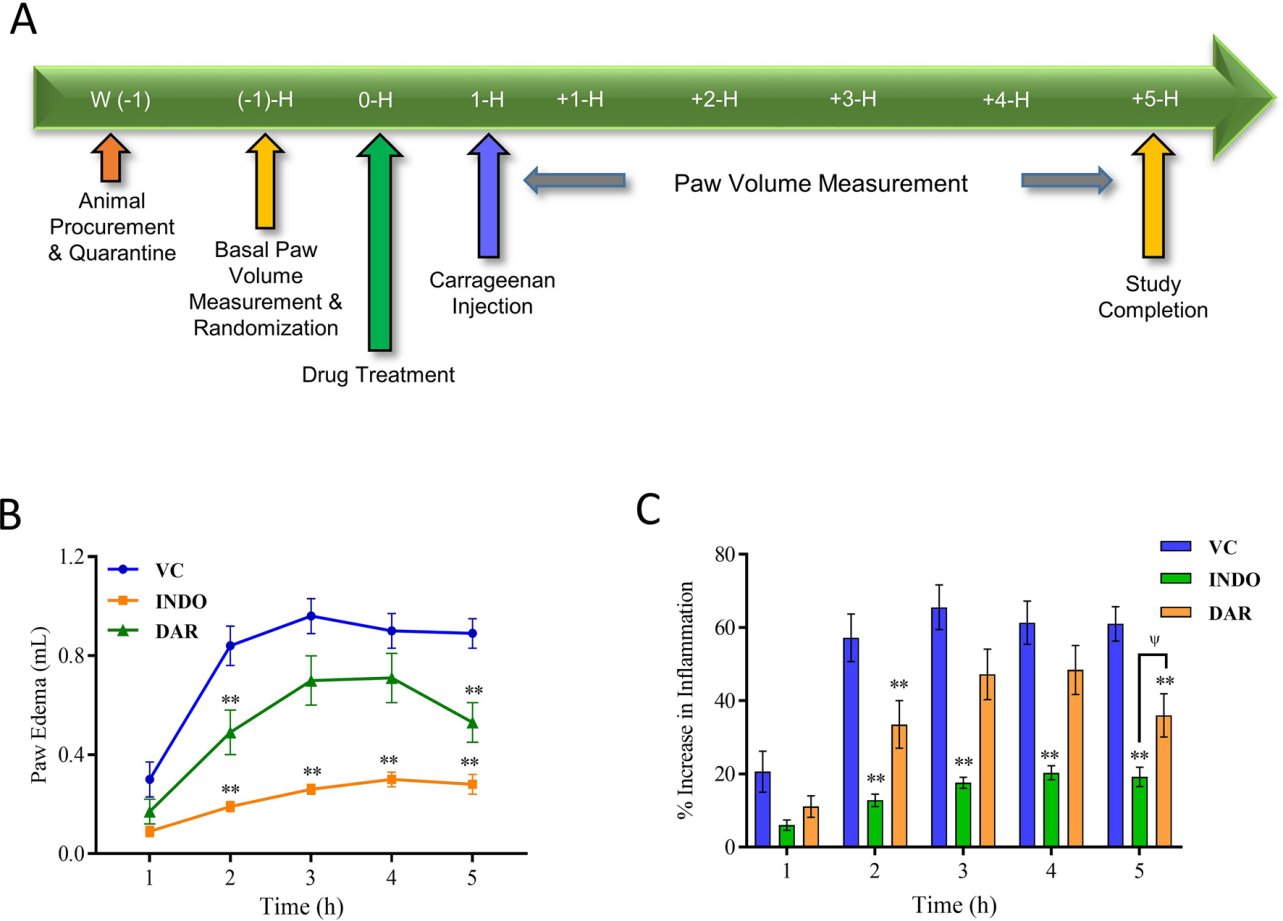
Effect of Divya Amvatari Ras (DAR) on carrageenan-induced paw edema. **(A)** Basal paw volume of male Wistar rats was measured and the rats were randomly assigned to normal control (NC), disease control (DC), and treatment groups. Treatment group rats were pre-treated with DAR (200 mg/kg rat) or indomethacin (INDO) (10 mg/kg rat) given orally followed by interplanar injection of carrageenan (CA) after 1 h, and paw volume was measured as a parameter for 5 h. **(B)** Paw volume was measured in the CA stimulated rats and showed an increase in the paw edema up to 5 h in the vehicle control (VC) rats. Treatment with DAR or INDO significantly reduced the paw edema. **(C)** Percentage increase in the inflammation from basal level showed that both the DAR and INDO treatments significantly reduced the paw. However, anti-inflammatory effects were higher in the INDO-treated animals. Values in the results are mean ± SEM. A two-way analysis of variance (ANOVA) followed by Neuman–Newman–Keuls multiple-comparison test was used to calculate statistical difference in VC versus INDO or DAR treatment (p-value ** < 0.01), and comparison between INDO and DAR treatments [p-value ψ > 0.05 (non-significant)].

Arthritis was induced in the Balb/c mice by intraperitoneal (I.P.) injection of a cocktail of five monoclonal antibodies to type II collagen (C-Ab) [1.5 mg in phosphate buffer saline (PBS) solution/mouse; day 0] followed by I.P. injection of 50 μg of LPS (from *Escherichia coli* strain 011B4; in a sterile normal saline) on day 3 (**Figure 1A**). The normal control (NC) animals received an equal volume of PBS only. On day 4, disease-induced animals were selected and randomized into four different groups of five animals each for treatments:

*Group I:* NC (non-induced: PBS + 0.25% Na-CMC p.o.; every day for 2 weeks)
*Group II:* Disease control (C-Ab + 0.25% Na-CMC p.o.; every day for 2 weeks)
*Group III:* C-Ab + MTX (0.38 mg/kg p.o.; every alternate day for 2 weeks)
*Group IV:* C-Ab + DAR (454 mg/kg p.o.; every day for 2 weeks)

The vehicle or MTX or DAR treatment was initiated from day 4 and continued for the next 2 weeks.

#### Assessment of Severity of Arthritis

The severity of arthritis in each mouse paw was scored every alternate day in a blinded manner according to the marginally modified method of Moore et al. and Khachigian on a 0–4 scale, as follows: 0 = normal; 1 = mild redness, slight swelling of ankle or wrist; 2 = moderate swelling of ankle or wrist; 3 = severe swelling, including some digits, ankle, and foot; 4 = maximally inﬂamed ankle and foot (Khachigian, 2006; Moore et al., 2014). The maximum possible score for each mouse was calculated as 16 in this study design. Mice paw and ankle thickness were measured using digital Vernier caliper (Mitutoyo, Japan) on days 0, 2, 4, 8, 12, and 16 for paw thickness, and 0, 2, 5, 9, 13, and 17 for ankle thickness. The paw and ankle joint edema was calculated by subtracting 0 h (basal) thickness from the respective thickness and post-treatment thickness on each day of the study.

#### Assessment of Pain Behaviors

##### Randall Selitto Pressure Test

The Randall–Selitto pressure test was performed to measure static hyperalgesia in animals according to the modified methods of Basile et al. (2007). The pain response as paw withdrawal threshold (PWT) to the mechanical stimulus was determined with a Randall and Selitto device on days 0, 2, 6, 10, and 14 after 1 h of vehicle or MTX or DAR administration. The PWT was defined as the force applied to the dorsal surface of the hind paw that causes the mouse to vocalize or withdraw the paw. A limit of 25 g was set to avoid tissue damage. From each mouse, an average of three readings with a gap of 5 min was recorded and analyzed in a blinded manner by a researcher unknown to the treatment conditions.

##### Hot Plate Test

Thermal hyperalgesia was assessed using a hot plate test as described by the method of Chagasa et al. (2017), with minor modifications. All the animals were placed into the Perspex cylinder of the hot plate (Ugo Saile, Italy) maintained at 55.0 ± 0.5°C, and time to discomfort reaction (licking paws or jumping) was recorded as response latency. The hot plate test was performed on days 0, 2, 7, 11, and 15, after 1 h of test article administration. A maximal cut-off point of 20 s was set to avoid any possible accidental paw damage. From each mouse, an average of three readings with a gap of 5 min was recorded and analyzed in a blinded manner by a researcher unaware to the treatment conditions.

#### Radiological Analysis

X-ray analysis was used to assess the osteo-morphology of mice hind limbs. At the end of the study, all the animals were humanely euthanized; left hind limbs were isolated and processed for X-ray images using an X-ray device (Siemens Heliophos-D Germany) at 40 kV and exposition of 0.01 s (at the Department of Radiology, Patanjali Ayurveda Hospital, Haridwar, India). The radiological analysis was done in a blinded manner by an external veterinary radiologist. Scoring of the abnormalities such as swelling of the soft tissue around the joints, periosteal hypertrophy, narrowing of the joint space, peri-articular osteoporosis, bone erosions, and any other lesions was done on the severity level: 0 = normal; 1 = slight; 2 = moderate; and 3 = severe (Nishikawa et al., 2003). Knee and ankle joints were analyzed separately.

#### Histological Examination

The right limb was harvested from humanely euthanized mice, fixed in 10% buffered neutral formalin, decalcified in 10% formic acid for 4 days, embedded in paraffin wax, sliced in solid sections of 3- to 5-µm thickness, and stained with hematoxylin and eosin (H&E) for general evaluation and Safranin O dye for specific assessment of cartilage damage. Blinded examination of histological slides was performed by an external veterinary pathologist to minimize bias. The severity of microscopic arthritic changes (enlargement in synovial lining cell layer, synovial hyperplasia, synovial vascularity, infiltration of inflammatory cells, pannus formation, cartilage erosion, and bone erosion) was evaluated in H&E-stained slides using the following grades: 0 = no abnormality detected; 1 = minimal (<1%); 2 = mild (1–25%); 3 = moderate (26–50%); 4 = marked (51–75%); 5 = severe (76–100%). Distributions of the lesions were recorded as focal, multifocal, and diffuse. Similarly, the severity of the cartilage degradation was scored as 0 = no apparent change; 1 = superficial fibrillation of articular cartilage; 2 = defects limited to uncalcified cartilage; 3 = defects extend into calcified cartilage; and 4 = exposure of subchondral bone at the articular surface. Scoring of knee and ankle joints was recorded separately. Images of the histological slides (H&E and Safranin O) were captured at low (⳨×100) and high (⳨×400) magnifications using an Olympus Magnus microscope camera and processed by Olympus MagVision image analysis software.

### Inductive Coupled Plasma–Mass Spectroscopy (ICP-MS) Analysis of Bone Tissue and Blood Serum

Bone tissue samples were collected from the femoral region of the humanely euthanized animals, and all muscular traces were removed. Samples were dried overnight at 37°C and were cut into a small piece with weight approx. 27–30 mg. An acid mixture of HNO_3_, H_2_O_2_, and HCl was prepared, and the bone samples were suspended in it while shaking (Rasmussen et al., 2013). For blood serum Hg level analysis, we followed the protocol suggested by Price et al. (2011). Briefly, 50 μl of blood serum was mixed with 50 μl of concentrated nitric acid and left to digest overnight. Samples were then heated for 20 min at 90°C to complete the digestion. The volume of each sample was reduced to approximately 40–50 μl after digestion; then 1 ml of 1% (v/v) nitric acid diluent was added to each cell sample. The dissolved bones and serum samples were collected and analyzed using ICP-MS (**Agilent Technologies; Model 7800**) with the following parameters: nebulizer gas flow: ∼0.9 L/min, auxiliary gas flow: ∼1.2 L/min, plasma gas flow: ∼15 L/min, integration time: 1,000 ms, inductive coupled plasma radio frequency (ICP RF) power: 1,200 W, and mode: helium. The ICP-MS data were analyzed using **Mass Hunter software **(**Agilent Technologies, USA**).

### Blood Biochemistry

For analysis of the alanine aminotransferase (ALT) and aspartate aminotransferase (AST), the Balb/c mice blood serum was isolated and stored at −80°C till further use. Commercially available kits for ALT (AL 8304) and AST (AS 8306) were purchased from Randox, USA, and ran on RX Monaco technology platform (Randox, USA), as per the manufacturer’s instructions. For measuring the pro-inflammatory cytokine IL-6 levels in the blood serum, we used Peprotech mouse IL-6 standard 2,2′-Azino-bis(3-ethylbenzothiazoline-6-sulfonic acid) diammonium salt (ABTS) enzyme-linked immunosorbent assay (ELISA) Development kit (900-K16) and followed the manufacturer’s instructions. ELISA plates were read at 450 nm using an Envision microplate reader (Perkin Elmer).

### Cell Culture for *In Vitro* Experiments

Human monocytic (THP-1) cells were obtained from the American Type Culture Collection (ATCC) authorized cell repository, National Centre for Cell Sciences, Pune, India. THP-1 cells were cultured in RPMI-1640 containing 10% heat-inactivated fetal bovine serum (FBS), in the presence of 100 units/ml of penicillin/streptomycin, 1 mM sodium pyruvate, and 4 mM L-glutamine. Cells were grown at 37°C in a 5% CO_2_/air environment.

### Cell Viability Analysis

One gram of the powdered DAR was suspended in incomplete culture media (RPMI-1640) at 37°C for 2 h. The insoluble part was cleared by high-speed centrifuge at 14,000 RPM. The cleared fraction was filtered with 0.2-μm filter and stored at 4°C till further use. THP-1 cells were plated in a 96-well plate at the concentration of 10,000 cells per well in a 96-well plate. The cells were pre-incubated overnight and exposed to the DAR at the concentrations of 0.0, 0.20, 0.39, 0.78, 1.56, 3.12, 6.25, 12.5, 25, and 50 mg/ml for a period of 24 h. At the end of the time period, the exposure medium was removed and the cells were washed with 100 μl PBS; 100 μl of 0.5 mg/ml 3-(4,5-dimethylthiazol-2-yl)-2,5-diphenyltetrazolium bromide (MTT) was added to each well, and the plates were incubated for 3 h at 37°C. At the end of the exposure period, the dye was removed. One hundred microliters of DMSO was added, and the plates were placed on a shaker for 10 min. Absorbance of each well was then read using the PerkinElmer Envision microplate reader at 595-nm wavelength, and cell viability percentage was calculated.

### Cytokine Level Measurement

Human monocyte THP-1 cells (5 × 10^5^ cells/well) were seeded in 24-well culture plates. To study the cytokine modulations, media with different concentrations of DAR were added to the wells at concentrations of 0.10, 0.33, 1.00, 3.30, and 10.00 mg/ml. After 1-h treatment, 500 ng/ml LPS was added to the cells, except in control wells. Consumed media or cell supernatants were collected after 24 h to measure different cytokine levels such as TNF-α, IL-1b, and IL-6 using standard ELISA kits (BD Biosciences). Three biological and technical replicates were performed for each experiment according to the manufacturer’s instructions, and plates were read at 450 nm using Envision microplate reader (Perkin Elmer).

### Luciferase Reporter NFκB Gene Assay

THP-1 cells were transiently transfected with luciferase reporter vector with NFκB promoter sequence upstream of luciferase gene. Transfection was performed following the manufacturer’s instruction in a 96-well plate using Lipofectamine 3000 (Invitrogen, USA). Two days after transfection, the experiment was performed as described by Ishimoto et al. (2015) with the following modifications. Used media was replaced with media containing test compound and control. After 1 h, LPS was added at a concentration of 500 ng/ml where required and incubated further for 12 h. D-Luciferin salt (Perkin Elmer) at a final concentration of 150 μg/ml was added to the cells and incubated at 37°C, protected from light. Relative percentage changes in light emission intensity were measured from each well and calculated; LPS alone was measured as 100% activity of the NFκB reporter gene.

### Statistical Analysis

The data are expressed as mean ± standard error of the mean (SEM) for each experiment. Statistical analysis was done using GraphPad Prism version 7.0 software. A two-way analysis of variance (ANOVA) followed by Neuman–Newman–Keuls multiple-comparison test and one-way ANOVA followed by Dunnett’s test were used as applicable to calculate the statistical difference. For the histological and radiological score analysis, we performed Kruskal–Wallis non-parametric analysis. Values of p < 0.05 were considered statistically significant.

## Results

### Divya Amvatari Ras Reduced Inflammation in Wistar Rats

The anti-inflammatory efficacy of DAR was assessed using CA-stimulated Wistar rat model (**Figure 1A**). Intraplantar injection of CA in rats led to a time-dependent increase in paw volume that was maximal at 3 h followed by marginal decrease at 5 h (**Figure 1B**). Animals pre-treated with DAR at 200 mg/kg displayed a significant (p < 0.01) reduction in the induction of paw edema at 2 and 5 h than did the vehicle control (VC) group (**Figure 1B**). The DAR demonstrated maximum anti-inflammatory activity as 48.7 ± 12.4%, 41.0 ± 11.2%, and 40.8 ± 9.2% at 1, 2, and 5 h, respectively (**Figure 1C**). The standard of care drug, INDO, at 10 mg/kg showed prominent (p < 0.01) anti-inflammatory activity at all time points except the 1-h time point.

### DAR Showed Anti-Arthritic Activity in CAIA CD-1 Mice

**Figure 2:**
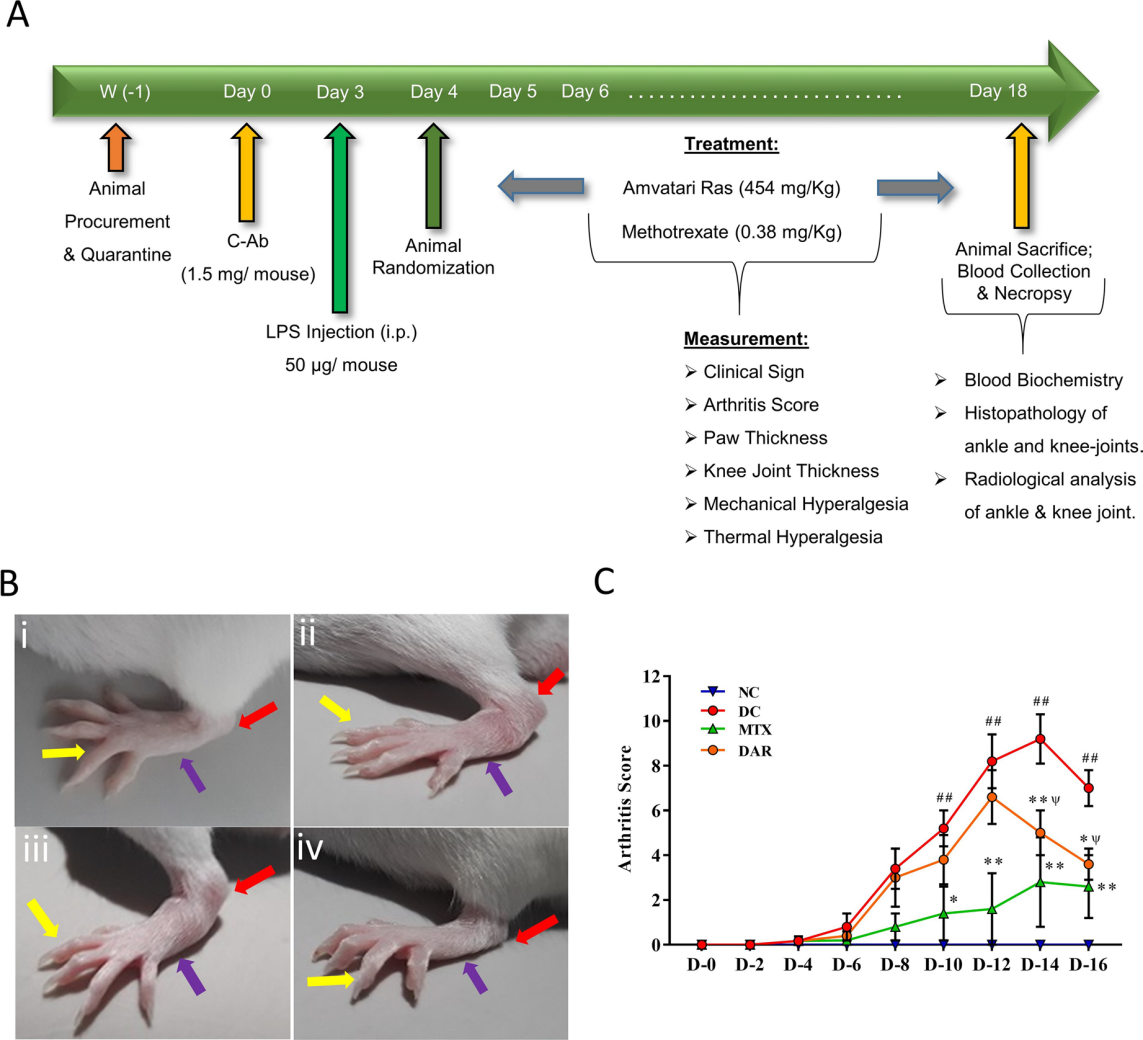
Induction of rheumatoid arthritis (RA) in Balb/C mice using collagen antibody (C-Ab) cocktail and modulation by DAR. **(A)** Male Balb/c mice were intraperitoneally treated with 1.5 mg/mouse dose of anti-collagen antibody (C-Ab) cocktail on day 0 and with 50 µg/mouse of bacterial lipopolysaccharide (LPS) on day 3. On day 4, all the animals were randomly assigned to the NC, DC, and treatment groups. The NC and DC animals were treated with sodium carboxymethyl cellulose (Na-CMC). The treatment group animals were given an oral dose of DAR (454 mg/kg) given every day for 2 weeks and standard reference drug Methotrexate (MTX; 0.38 mg/kg) given every alternate day for 2 weeks. Physical and clinical parameters were measured every day throughout the experimental duration. **(B) (i)** NC animal showed normal digits (yellow arrow), foot (blue arrow), and ankle (red arrow); **(ii)** development of digits, foot, and ankle edema in the DC animal; **(iii)** reduction of the digits, foot, and ankle edema in collagen antibody-induced arthritis (CAIA) mice treated with DAR; and **(iv)** MTX. **(C)** Arthritis score analysis showed an increase in arthritis scores in DC animals as compared to the NC animals. Treatment of the CAIA mice with DAR and MTX significantly reduced the arthritis score. Values in the results are mean ± SEM. A two-way analysis of variance (ANOVA) followed by Neuman–Newman–Keuls multiple-comparison test was used to calculate statistical difference on individual days in NC versus DC (p-value ## < 0.01), MTX or DAR treatment versus DC (p-value * < 0.05; ** < 0.01), and comparison between MTX and DAR treatments [p-value ψ > 0.05 (non-significant)].

Anti-arthritic efficacy of DAR was studied using collagen antibody cocktail (C-Ab) induced arthritis (CAIA) mouse model (**Figure 2A**). The disease control (DC) mice showed development of edema in ankle, feet, and digits, as compared to the NC animals [**Figure 2B(i) and (ii)**]. CAIA animals orally treated with DAR and MTX showed a clear reduction of the pedal region swelling [**Figure 2B(iii) and (iv)**]. Arthritis score analysis showed significantly elevated score in the DC mice between days 10 and 16 (p < 0.01) (**Figure 2C**). DAR treatment of CAIA animals showed considerable reduction of arthritis on days 14 (p < 0.01) and 16 (p < 0.05). It is noteworthy that the effects shown by the DAR on days 14 and 16 were statistically non-significant as compared to MTX.

### DAR Treatment Modulated Pain Behaviors

**Figure 3:**
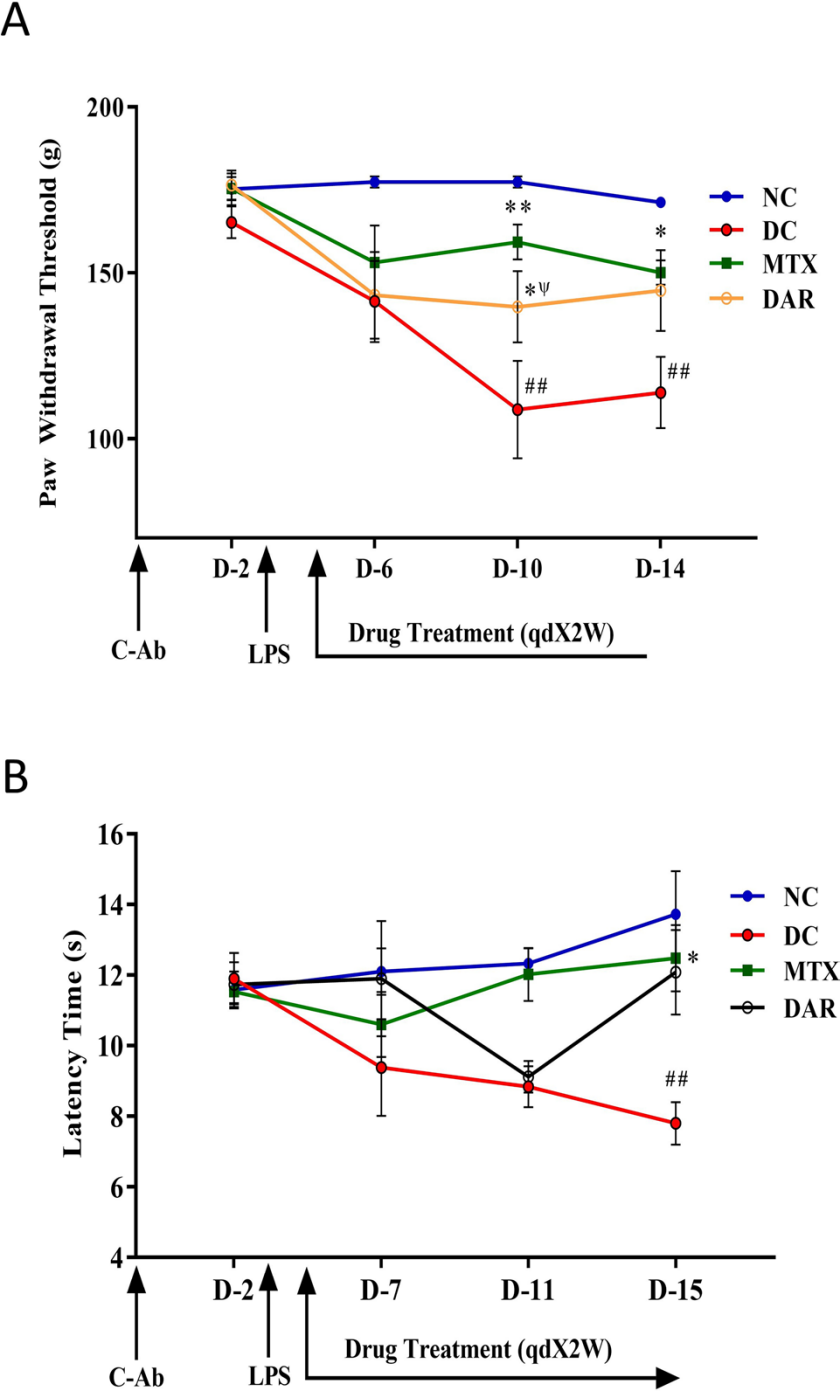
Pain sensitivity modulation by DAR treatment. **(A)** Paw withdrawal threshold was measured using Randall Selitto (mechanical hyperalgesia) parameter. The results showed a significant decrease in the paw withdrawal threshold following the onset of RA in the collagen-antibody cocktail (C-Ab) and LPS stimulated DC animals as compared to the NC. Reduction in mechanical hyperalgesia was observed following the treatment of collagen-antibody-induced arthritis mice with DAR and MTX. **(B)** Thermal hyperalgesia tested using the hot plate test was found to be increased in the DC animals following exposure to C-Ab and LPS as compared to the NC animals. This was found to recover back to normal levels following treatment with DAR and MTX. Values in the results are mean ± SEM. A two-way analysis of variance (ANOVA) followed by Neuman–Newman–Keuls multiple-comparison test was used to calculate statistical difference on individual days in NC versus DC (p-value # < 0.05; ## < 0.01), MTX or DAR treatment versus DC (p-value * < 0.05), and comparison between MTX and DAR treatments [p-value ψ > 0.05 (non-significant)].

The severity of the RA disease has been associated with stimulation of chronic neuroinflammation and hyperalgesia or increased pain sensitivity (Morris et al., 1997; Fuggle et al., 2014). Mechanical hyperalgesia was analyzed using Randall-Selitto test (**Figure 3A**). CAIA mice showed a significant decrease in PWT than did the NC animals, on days 10 and 14 (p < 0.01) (**Figure 3A**). CAIA mice treated with DAR demonstrated considerable increase in PWT as 139.7 ± 10.7 and 144.6 ± 12.2 g on days 10 and 14, respectively. However, statistically significant changes (p < 0.05) were only observed on day 10. The reference drug MTX showed significant increase in PWT on day 10 (159.3 ± 5.2 g; p < 0.01) and on day 14 (150.0 ± 3.6 g; p < 0.05). Importantly, effects shown by DAR were found to be comparable to those of MTX (p > 0.05) on day 10.

The effect of DAR on disease induced pain sensitivity was also analyzed using hot plate test (as shown in **Figure 3B**). CAIA mice showed considerable decrease in latency time at day 7 (9.4 ± 1.4 s), 11 (9.9 ± 0.6 s), and 15 (7.8 ± 0.6 s; p < 0.05), as compared to NC animals. Further, treatment of DAR displayed noticeable increase in latency time at days 7, 11, and 15. However, significant (p < 0.05) effect of DAR was observed at day 15 (12.1 ± 1.2 s). Similarly, reference drug MTX exhibited prominent effect on decrease in pain sensitivity with rise in latency time at day 15 (12.5 ± 0.9 s) (p < 0.05).

### DAR Treatment Reverses the Arthritic Bone Damages

**Figure 4:**
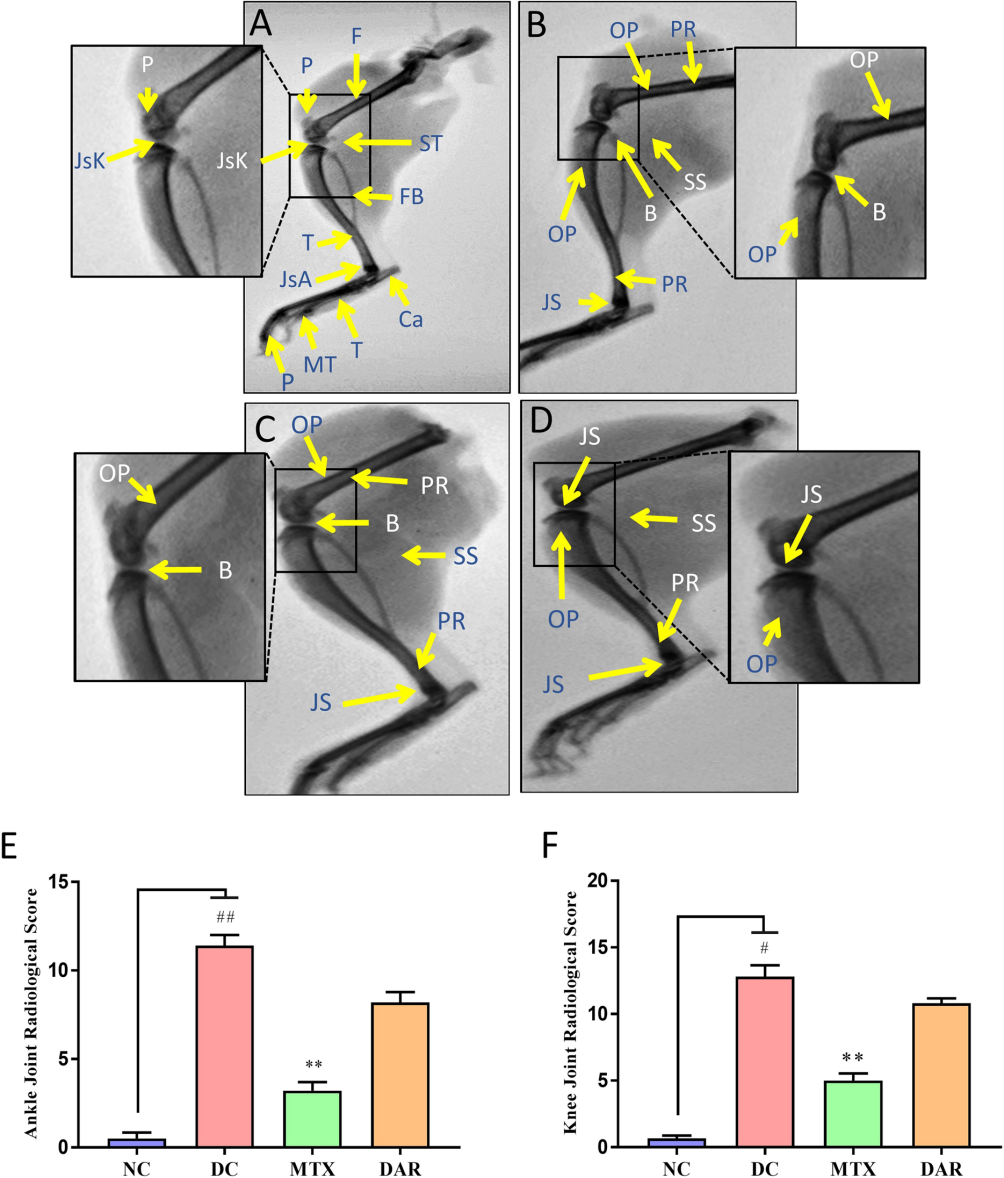
Radiological analysis of RA mice limbs: X-ray images of the hind legs were taken. **(A)** NC animal showing normal morphology of bones, *viz.*, tibia (T), fibula (Fb), calcaneum (Ca), patella (P), femur (F), tarsals (T), metatarsals (MT), and phalanges (P). Normal joint space with healthy cartilage at the knee joint (JsK) and ankle joint (JsA) and periarticular soft tissue (ST); inset showing knee joint. **(B)** DC animal showing periosteal reaction/hypertrophy (PR), bone erosion (B), soft tissue swelling (SS), narrowed joint space (JS), and osteoporosis (OP); inset showing knee joint. **(C)** Methotrexate (MTX) treated CAIA animal showing PR, B, SS, JS, and OP; inset showing knee joint. **(D)** CAIA animal treated with DAR showing PR, SS, JS, and OP; inset showing knee joint. **(E)** Ankle-joint radiological score showed significant arthritis-induced bone damages in the DC animals. Treatment of the CAIA animals with DAR and MTX considerably reduced arthritis-induced bone damages. **(F)** Knee-joint radiological score showed results similar to those observed in the ankle joint of the CAIA animals. Non-parametric Kruskal–Wallis analysis was used to calculate the statistical difference in NC versus DC (p-value # < 0.05; ## < 0.01), MTX or DAR treatment versus DC (p-value * < 0.05; ** < 0.01).

Radiological scoring was performed using anonymized X-ray images for documenting tissue edema and bone injuries in the NC, DC, and DAR and MTX treated CAIA mice (**Figure 4A–D**). Radiological images indicated development of periosteal reaction/hypertrophy, bone erosion, soft tissue swelling, narrowed joint space, and osteoporosis in the DC animals (**Figure 4B**). CAIA animal treated with DAR and MTX showed reduction in periosteal reaction/hypertrophy, soft tissue swelling, narrowed joint space, and osteoporosis (**Figure 4C and D**). Ankle and knee-joint radiological score showed significant arthritis-induced bone damages in the DC animals. Treatment of the CAIA animals with DAR and MTX (p < 0.01) considerably reduced arthritis-induced bone damages in the ankle-joint and knee-joint regions (**Figure 4E and F**). Compared with the DAR, treatment of the CAIA animals with MTX showed slightly higher efficacy in reducing soft tissue and bone damages in the ankle- and knee-joint regions (**Figure 4E and F**).

### DAR Treatment Attenuated Arthritis Symptoms and Histopathological Scores

**Figure 5:**
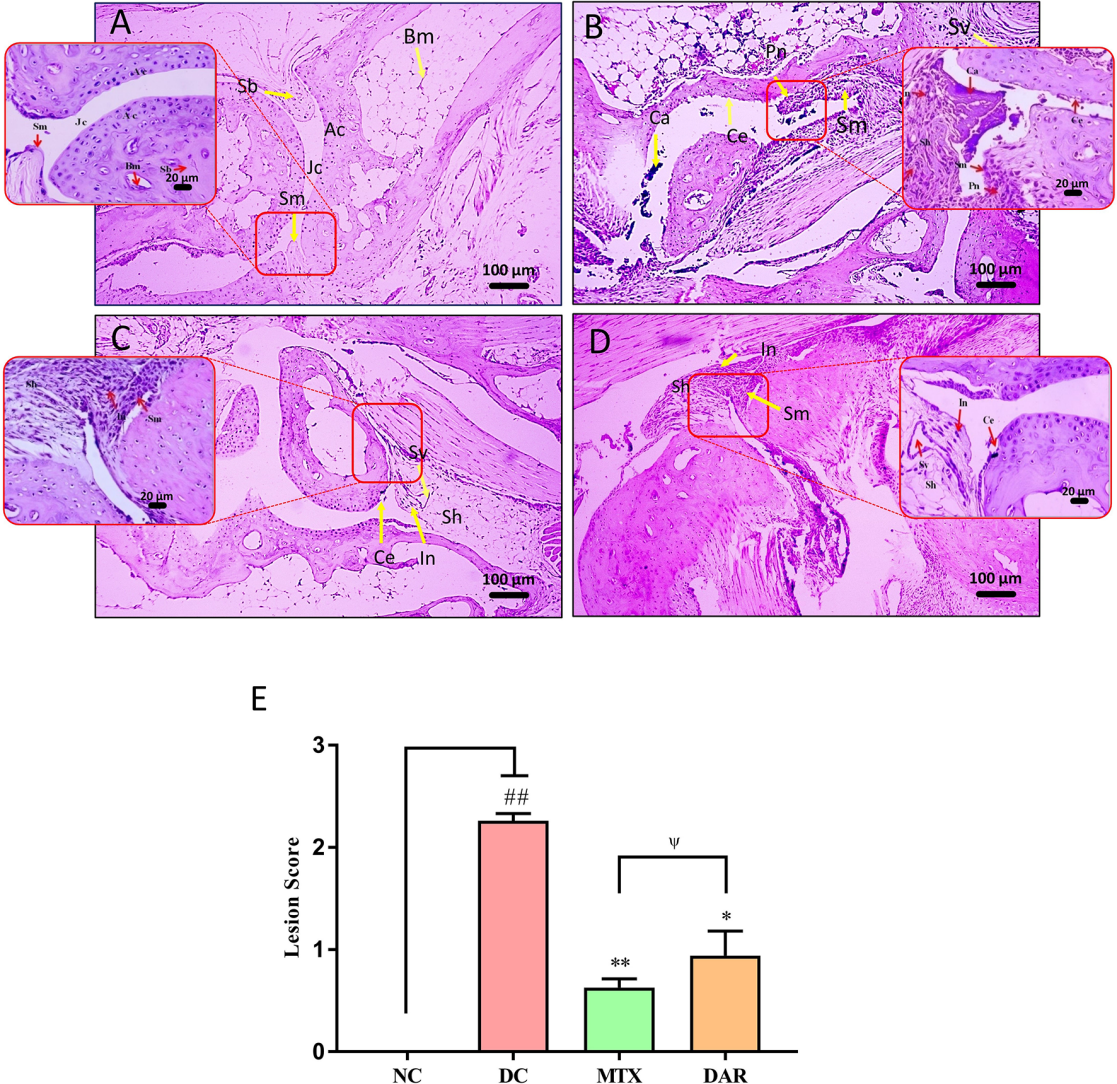
H&E stained histopathological analysis of ankle joints. Histopathological images of H&E stained ankle joints taken at low (100×) and high (400×) magnification showing: **(A)** NC animal showing synovial membrane (Sm), spongy bone (Sb), bone marrow (Bm), joint cavity (Jc). **(B)** Collagen-antibody and lipopolysaccharide stimulated DC animal showing moderately enlarged synovial membrane (Sm), hyperplastic synovium (Sh), increased synovial vascularity (Sv), calcinosis (Ca), inflammation (In), pannus formation (Pn), and cartilage erosion (Ce). **(C)** CAIA animals treated with DAR showed mildly enlarged synovial membrane (Sm), hyperplastic synovium (Sh), and presence of inflammatory cells (In). **(D)** CAIA animals treated with MTX showed enlarged hyperplastic synovium (Sh), inflammation (In), increased synovial vascularity (Sv), and cartilage erosion (Ce). **(E)** Total lesion score measurement indicated a decrease in total inflammatory lesion score in the CAIA animals following treatment with DAR and MTX. Non-parametric Kruskal–Wallis analysis was used to calculate the statistical difference in NC versus DC (p-value ## < 0.01), MTX or DAR treatment versus DC (p-value * < 0.05; ** < 0.01), and comparison between MTX and DAR treatments [p-value ψ > 0.05 (non-significant)].

Histopathological analysis of the DC animal group ankle joint, post 18 days’ onset of RA disease, showed moderate enlargement of ankle-joint synovial membrane, hyperplastic synovium, increased synovial vascularity, presence of inflammation, and bone and cartilage erosion, as compared with NC animal (**Figure 5A and B**). Significantly, high lesion score (p < 0.01) was observed in the ankle joint of CAIA animals (**Figure 5E** and **Supplementary Figure 2A**–**G**). Following DAR treatment of the CAIA mice, a significant (p < 0.05) reduction in the ankle-joint lesion scores was observed, indicating reduced inflammation (**Figure 5C–E**). Both DAR and MTX displayed similar efficacies in reducing ankle-joint inflammation (**Figure 5C–E**).

**Figure 6:**
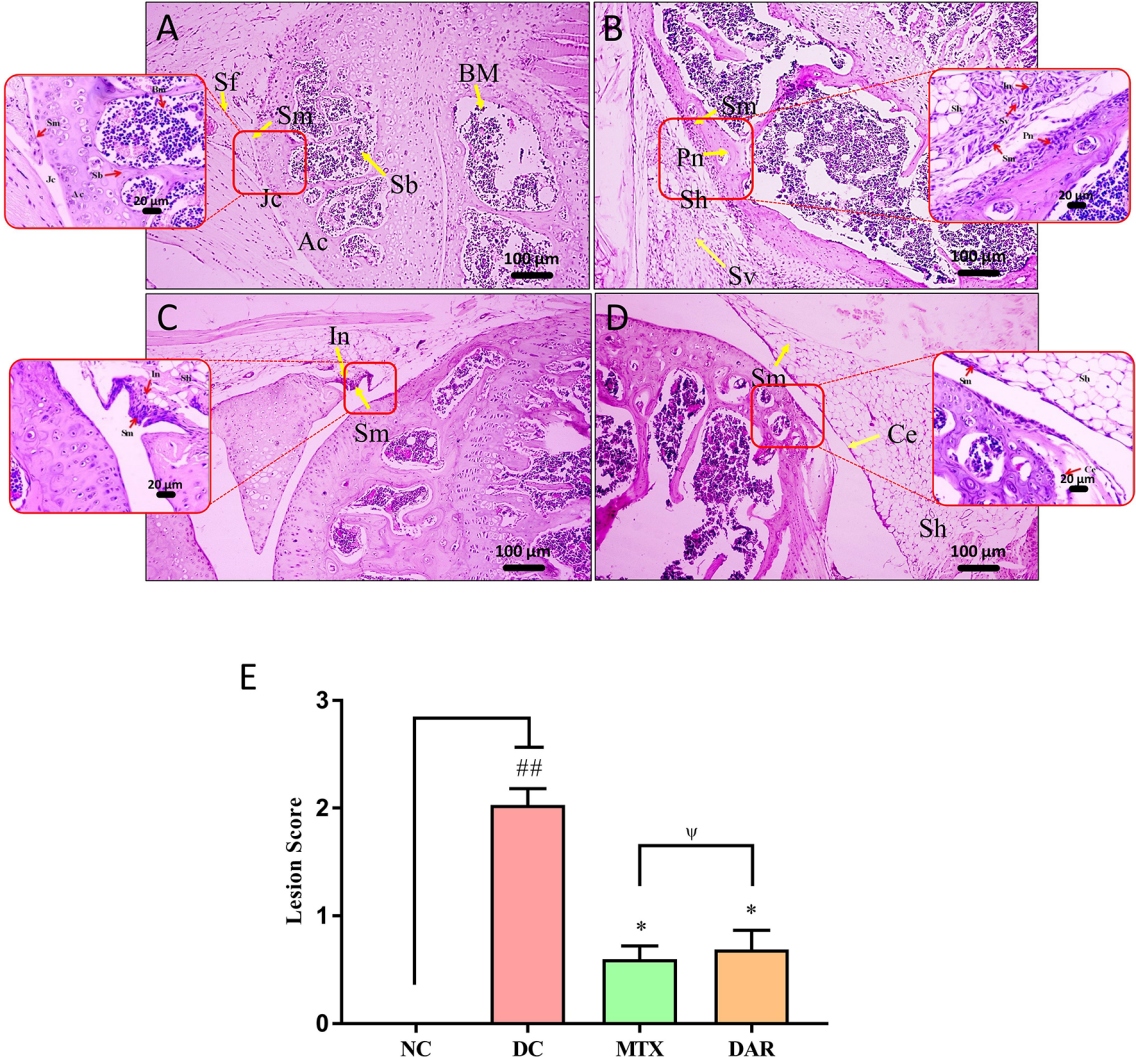
H&E stained histopathological analysis of knee joints. Histopathological images of H&E stained knee joints taken at low (100×) and high (400×) magnification showing: **(A)** NC animal representing articular cartilage (Ac), synovial membrane (Sm), spongy bone (Sb), bone marrow cells (Bm), and joint cavity (Jc). **(B)** C-Ab and LPS stimulated DC animal showing moderately enlarged synovial membrane (Sm), hyperplastic synovium (Sh), increased synovial vascularity (Sv), inflammation (In), and Pannus formation (Pn). **(C)** Treatment of the CAIA mice with DAR showed mildly enlarged Sm, Sh, and cartilage erosion (Ce). **(D)** Treatment of the CAIA mice with MTX showed mildly enlarged Sm, Sh, increased Sv, and induction of inflammation (In). **(E)** Total lesion score analysis showed that the treatment of CAIA mice with DAR or MTX showed a significant reduction in the lesion score. Non-parametric Kruskal–Wallis analysis was used to calculate the statistical difference in NC versus DC (p-value ## < 0.01), MTX or DAR treatment versus DC (p-value * < 0.05; ** < 0.01), and comparison between MTX and DAR treatments [p-value ψ > 0.05 (non-significant)].

Analysis of knee-joint lesion scores in the CAIA animals showed significant (p < 0.01) induction of inflammatory lesions through the development of moderately enlarged synovial membrane, hyperplastic synovium-increased synovial vascularity, calcinosis, inflammation, pannus formation, and cartilage erosion (**Figure 6A–E**). Treatment of the CAIA mice with DAR and MTX (p < 0.05) reduced knee-joint synovial membrane inflammation, pannus formation, cartilage, bone erosions, and total lesion score (**Figure 6E**; **Supplementary Figure 3A**–**G**). A similar efficacy was detected for both DAR and MTX in reducing knee-joint inflammation-associated lesion scores (**Figure 6E**).

**Figure 7:**
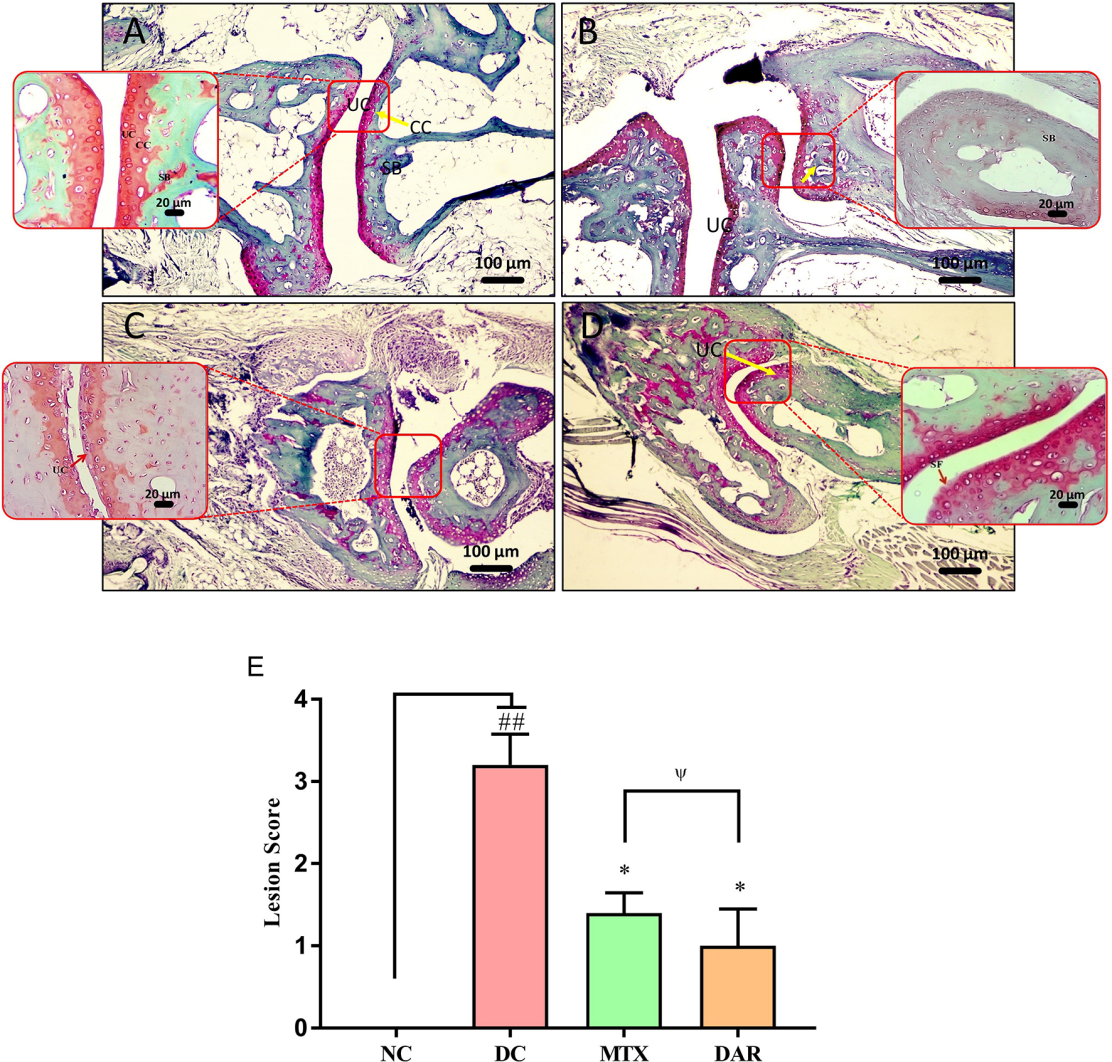
Effect of DAR treatment on articular cartilage erosion of ankle joints. Low (100×) and high (400×) magnification images of safranin-O stained sections of the ankle joints showing: **(A)** Normal histology in the untreated Balb/c mice showing uncalcified cartilage (UC), calcified cartilage (CC), and subchondral bone (SB). **(B)** Collagen-antibody stimulated DC animal showing cartilage degradation extending up to SB. **(C)** CAIA mice treated with DAR showing cartilage degradation till the UC region. **(D)** CAIA mice treated with MTX showing cartilage degradation limited till the UC region. **(E)** Total lesion score analysis indicated lesion development in the collagen-antibody stimulated mice (DC) that were significantly reduced following treatment with DAR or MTX. DAR and MTX showed similar efficacy in the treatment of collagen-antibody induced ankle-joint lesions. Values in the results are mean ± SEM. Non-parametric Kruskal–Wallis analysis was used to calculate the statistical difference in NC versus DC (p-value ## < 0.01), MTX or DAR treatment versus DC (p-value * < 0.05), and comparison between MTX and DAR treatments [p-value ψ > 0.05 (non-significant)].

**Figure 8:**
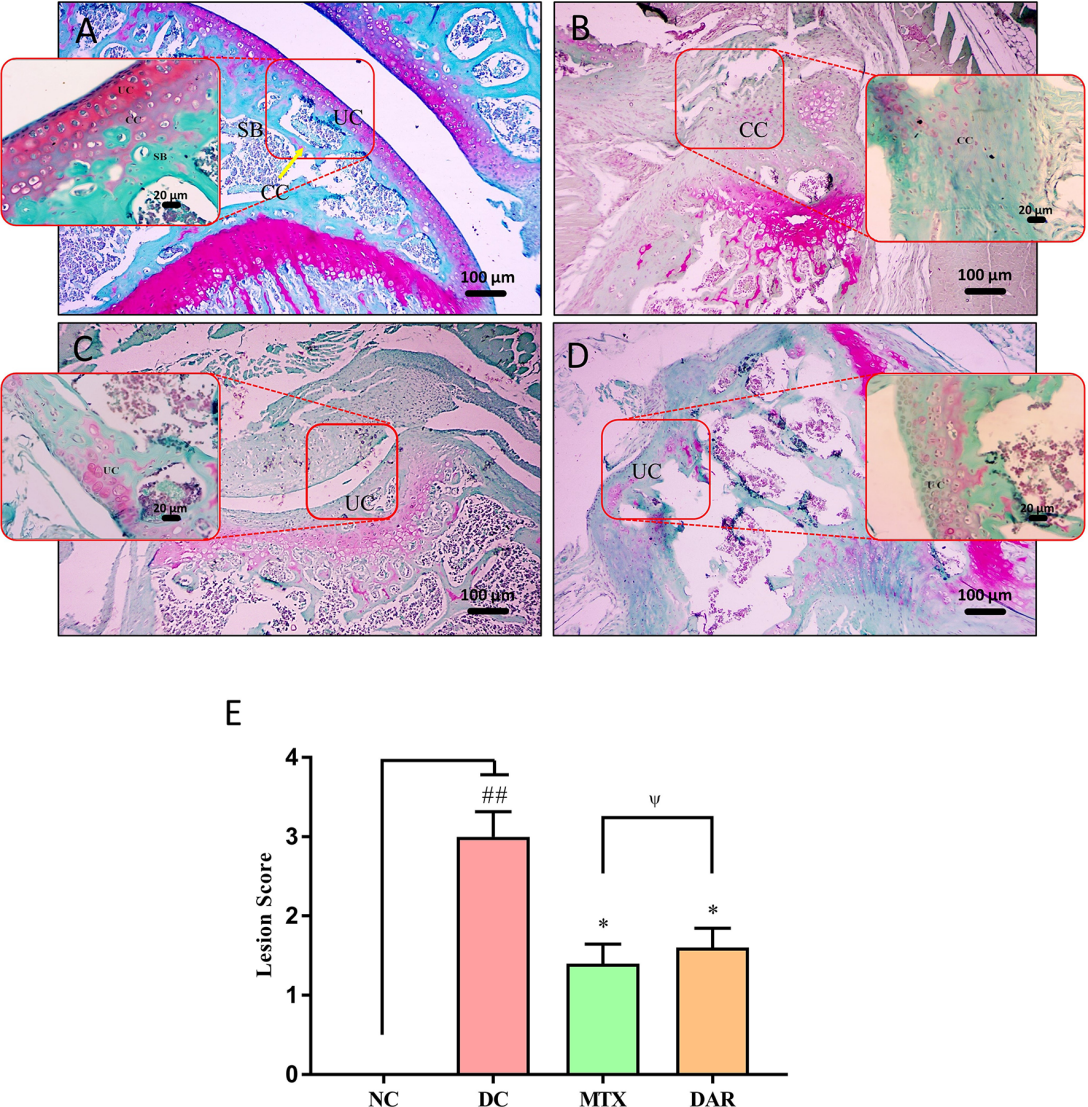
Effect of DAR treatment on articular cartilage erosion of knee joint. Low (100×) and high (400×) magnification images of safranin-O stained sections of the knee joints showing: **(A)** Normal knee-joint histology in the untreated Balb/c mice showing normal uncalcified cartilage (UC), calcified cartilage (CC), and subchondral bone (SB). **(B)** Collagen-antibody stimulated DC animal showing cartilage degradation extending up to the SB region. **(C)** Treatment of CAIA mice with DAR showing cartilage degradation till the UC region. **(D)** Treatment of the CAIA mice with MTX showed superficial fibrillation of the articular cartilage (FB) region. **(E)** Collagen-antibody stimulated mice showed an increase in total lesion score induction knee-joint cartilage region damages. Significant reduction in the lesion score was detected in the CAIA mice treated with DAR and MTX. Both DAR and MTX treatments showed similar efficacy. Values in the results are mean ± SEM. Non-parametric Kruskal–Wallis analysis was used to calculate the statistical difference in NC versus DC (p-value ## < 0.01), MTX or DAR treatment versus DC (p-value * < 0.05), and comparison between MTX and DAR treatments [p-value ψ > 0.05 (non-significant)].

Safranin “o” staining of the collagen matrix showed severe articular cartilage damage in the ankle and knee joints of the DC animal as compared to the healthy mice (**Figures 7A, B** and **8A, B**). Severe cartilage damage was observed in the ankle and knee joints of the CAIA mice extending up to the subchondral region bone (**Figures 7B** and **8B**). Damaged cartilage region represents severe inflammation and high catabolic reaction from the mature chondrocytes. Total lesion score analysis indicated significantly high score (p < 0.01) in the ankle and knee joints of the DC animals (**Figures 7E** and **8E**). Treatment of CAIA animals with DAR or MTX individually showed limited cartilage damages in the ankle- and knee-joint regions extending up to the uncalcified cartilage region. The results indicated that both the DAR and MTX showed a beneficial effect in inhibiting RA induced cartilage layer destruction (**Figures 7C, D** and **8C, D**). Lesion score analysis in the ankle and knee joints further confirmed the histological observations by showing a significant reduction in the mean lesion score (p < 0.05) as compared to the DC animals (**Figures 7E** and **8E**). Efficacy of both the DAR (p < 0.05) and MTX (p < 0.05) treatments was found similar in reducing ankle- and knee-joint lesions (**Figures 7E** and **8E**).

### No Hg Bioaccumulation in Repeated Dosing of DAR in CAIA Mice

**Figure 9:**
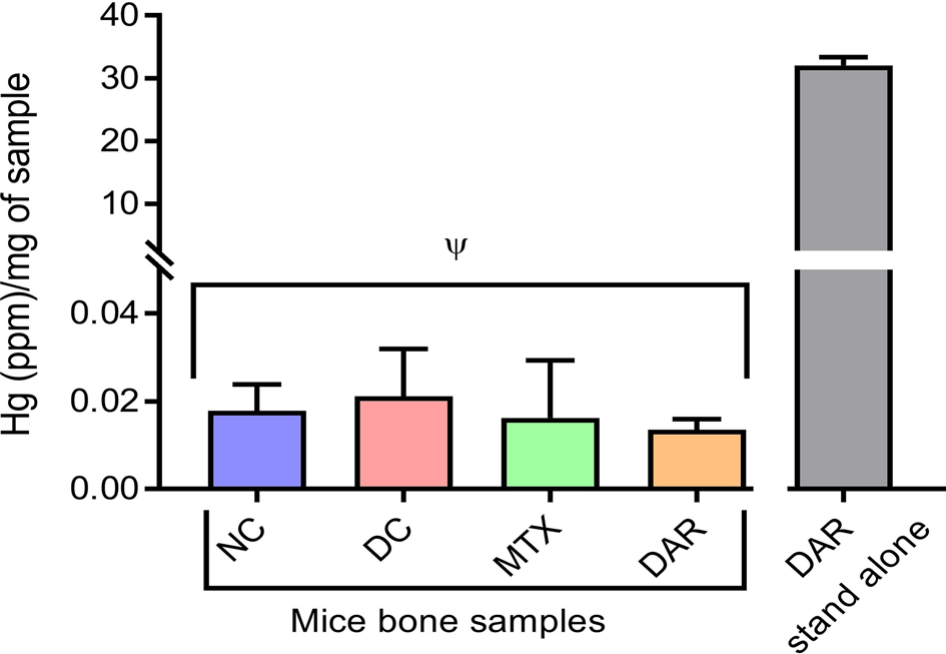
Mercury (Hg) levels in the bone tissue of NC, DC, and treated (MTX and DAR) animals. The amount of Hg present in the DAR stand-alone tablet was detected at 38.02 ppm/mg sample. Values in the results are mean ± SD. A one-way analysis of variance (ANOVA) followed by Dunnett test was used to calculate the statistical difference between NC *versus* DC, and MTX or DAR treatment *versus* DC. p-value ψ > 0.05 (non-significant).

Analysis for bioaccumulation of Hg in the bone tissue of CAIA mice treated with DAR was performed using ICP-MS technologies. Bone samples obtained from the femoral region of animals showed 0.013 ± 0.002 parts per million (ppm) Hg/mg of the bone tissue after 2-week treatment of DAR. This was statistically close to the Hg levels (0.017 ± 0.005 ppm/mg) detected in the bone tissue of the healthy NC animal (**Figure 9**). In addition, Hg levels in the blood serum of the NC, DC, DAR-treated, and MTX-treated CAIA mice were found to be below the detection limits of the ICP-MS equipment. In the DAR stand-alone sample, the level of Hg present was discovered at 32.08 ± 1.26 ppm/mg of DAR. These data indicated that Hg did not bioaccumulate in the bones and serum of animals during repeated treatment with DAR (**Figure 9**).

### DAR Treatment Reduced Serum IL-6 and ALT Levels

**Figure 10:**
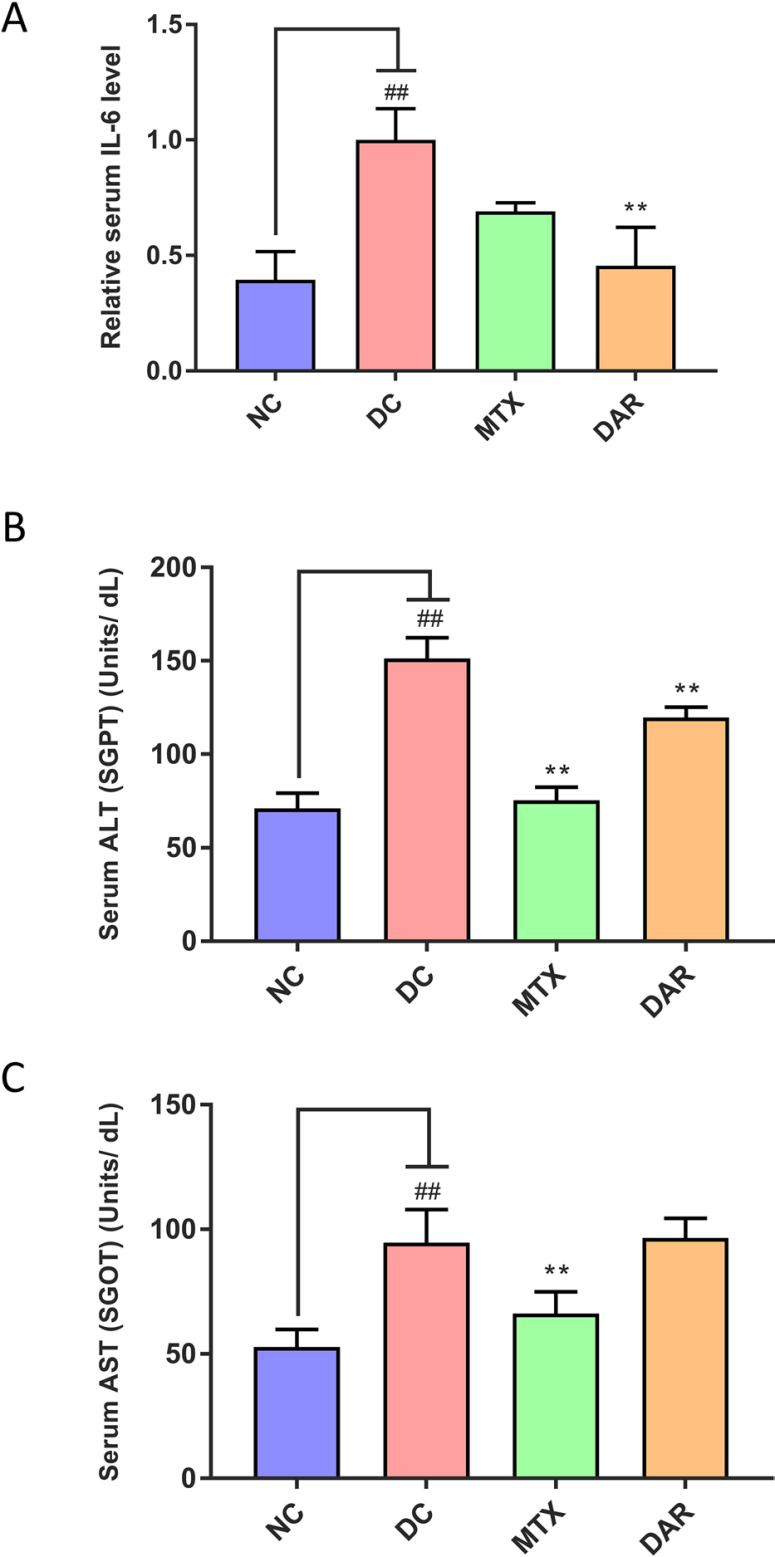
Biomarker measurement in blood serum. **(A)** Analysis of the serum IL-6 levels showed its induction in the collagen-antibody stimulated disease control (DC) Balb/c mice. Treatment of the collagen-antibody-induced arthritis mice with DAR and MTX led to a significant serum IL-6 inhibition as compared to the DC animals. **(B)** Measurement of liver enzyme alanine aminotransferase (ALT), also known as serum glutamate-pyruvate transaminase (SGPT), was done in the blood serum of the mice and showed an increase in the DC animals indicating the onset of liver damage as compared to the NC animals. Treatment of CAIA mice with DAR or MTX showed a significant reduction in the levels of ALT post-treatment. **(C)** Analysis of the aspartate aminotransferase (AST) also known as serum glutamic-oxaloacetic transaminase (SGOT) enzyme showed a substantial increase in the CAIA mice as compared to the NC mice. DAR treatment did not show any modulation in liver toxicity as compared to the DC animals; MTX treatment showed a significant reduction in the AST levels as compared to the DC animals. Values in the results are mean ± SEM. A one-way analysis of variance (ANOVA) followed by Dunnett’s multiple comparison t-test was used to calculate the statistical difference. Student’s unpaired t-test was used to calculate the statistical difference in comparison to MTX. P-value ## < 0.01 and ** < 0.01.

Pro-inflammatory cytokine, interleukin 6 (IL-6) elevation in the blood serum was studied in the CAIA mice (**Figure 10A**). Results indicated two-fold elevation in the IL-6 cytokine level in the DC animals from the basal level. Treatment of the CAIA animals with DAR or MTX led to considerable reduction in the C-Ab stimulated expression of IL-6 (**Figure 10A**). Amelioration of IL-6 release by the DAR treatment was found to be significant (p < 0.01) as compared to the DC animals.

Role of CAIA in inducing liver damages in the treated Balb/c mice and its amelioration through DAR and MTX treatments was analyzed in the blood serum enzymatic biomarkers ALT and AST (**Figure 10B and C**). DC animals following stimulation with C-Ab cocktail and LPS showed significantly high release of both ALT and AST (p-value < 0.01) (**Figure 10B and C**). Treatment of the CAIA animal with DAR significantly reduced the ALT levels (**Figure 10B**), while no reduction was observed for the AST enzymatic biomarker (**Figure 10C**). MTX treatment of the CAIA animals exhibited substantial decrease of both the ALT and AST biomarkers in the blood serum (**Figure 10B and C**). It is noteworthy that both DAR and MTX treatments did not induce any additional elevation of serum ALT and AST levels, in comparison with DC animals, suggesting that these treatments did not induce any gross level changes in the liver functions of CAIA Balb/c mice.

### Cyto-Safety of DAR in Human Monocytic THP-1 Cells

**Figure 11:**
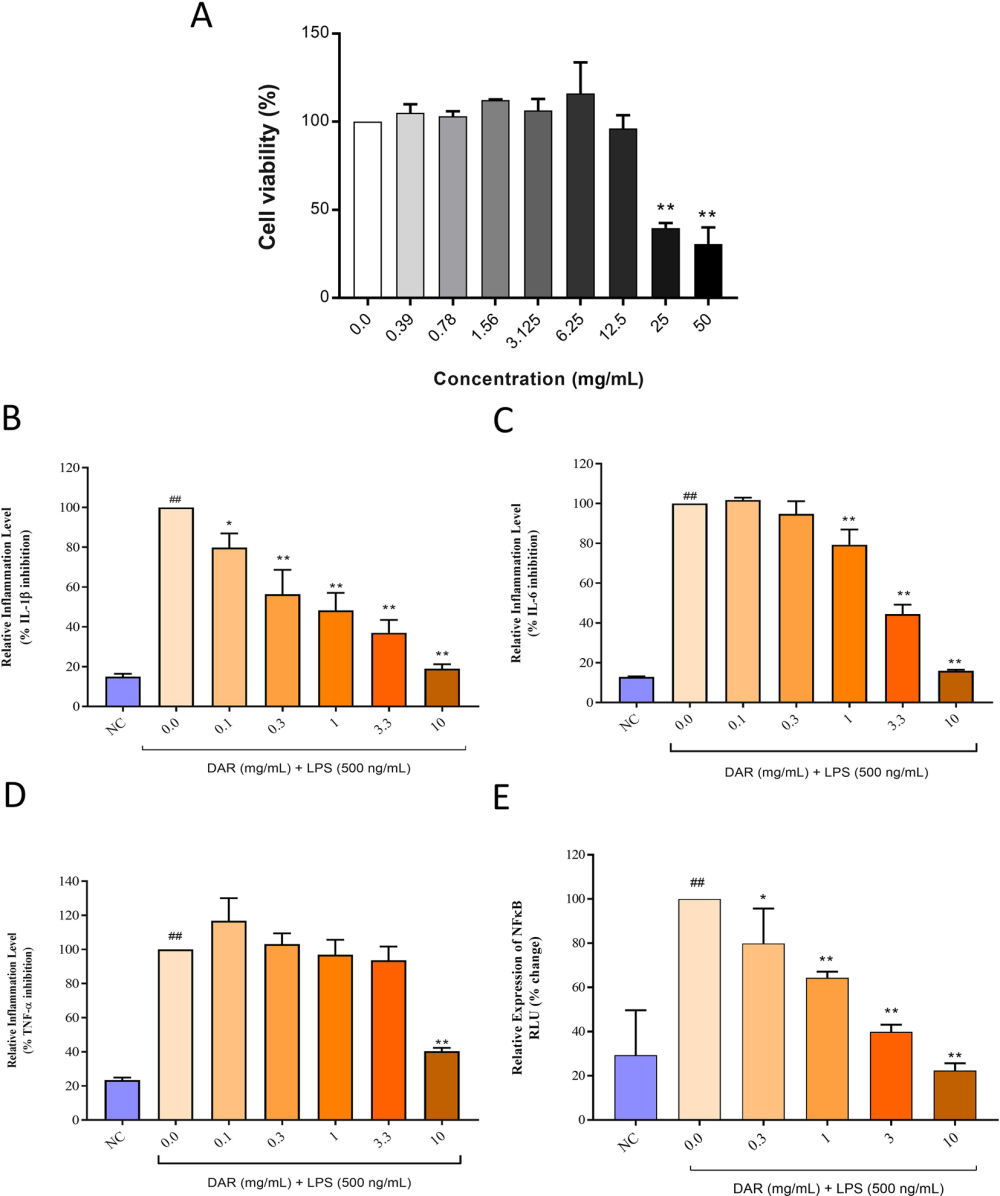
Cyto-safety and in vitro modulation of pro-inflammatory mediators by DAR. **(A)** THP-1 cells treated with varying concentrations of the DAR between 0 and 50 mg/ml showed induction of toxicity at dose >12.5 mg/ml. The inhibitory concentrations 20% and 50% were found at 14.70 and 32.19 mg/ml, respectively. Pro-inflammatory responses in the lipopolysaccharide (LPS) treated THP-1 cells showed stimulated release of the pro-inflammatory cytokines **(B)** IL-1β, **(C)** IL-6, and **(D)** TNF-α. Treatment of the THP-1 cells with varying concentrations of the DAR inhibited the production of the pro-inflammatory cytokines in a dose-dependent manner. **(E)** LPS stimulation in luciferase NFκB reporter gene transfected THP-1 cells led to high expression of NFκB protein. This elevation was reduced by DAR in a dose-dependent manner, up to the tested concentration of 10 mg/ml. Values in the results are mean ± SEM. A one-way analysis of variance (ANOVA) followed by Dunnett’s multiple-comparison test was used to calculate the statistical difference between normal control versus LPS only (p-value ## < 0.01), and DAR + LPS treatment versus LPS only (p-value * < 0.05; ** < 0.01).

Biocompatibility of DAR was investigated in human monocytic (THP-1) cells by measuring cell viability (**Figure 11A**). Results indicated that the DAR did not induce any loss of cell viability up to the measured concentration of 12.5 mg/ml. Based on the cell viability assay results, we calculated inhibitory concentration 20% (IC_20_) and IC_50_ for DAR at 14.7 and 32.19 mg/ml, respectively. Statistically significant toxicity was detected at the DAR concentrations of 25 and 50 mg/ml (p-value < 0.01) (**Figure 11A**). It is worthwhile to note that MTX has been reported to have an IC_50_ in the range of 30 μM, equivalent to 13.6 μg/ml tested across several cell lines (Xie et al., 2016).

### DAR Inhibited the Release of Pro-Inflammatory Cytokines in Human Monocytic THP-1 Cells

For elucidating the mechanism of action of the DAR under *in vitro* conditions, we studied the modulation of IL-1β, IL-6, and TNF-α cytokines in LPS stimulated human monocytic (THP-1) cells (**Figure 11B–D**). These pro-inflammatory cytokines were found to be upregulated in the THP-1 cells stimulated with 500 ng/ml of LPS. Treatment of the LPS-stimulated cells with the DAR between the concentration of 0.1 and 10 mg/ml significantly reduced the levels of IL-1β cytokine in a dose-dependent manner (**Figure 11B**). The highest level of IL-1β reduction was detected at the DAR dose of 10 mg/ml (p < 0.01) (**Figure 11B**). Similarly, both the IL-6 and TNF-α cytokines were found to be significantly reduced in the DAR (0.1–10 mg/ml) treated LPS stimulated THP-1 cells (**Figure 11C and D**). The highest reduction of TNF-α cytokine release in the LPS stimulated THP-1 cells was detected at the DAR dose of 10 mg/ml (p < 0.01) (**Figure 11D**).

Stimulation of THP-1 cells with LPS led to an increased expression of the upstream pro-inflammatory protein nuclear factor-κB (NFκB) (**Figure 11E**). This elevation was five-folds higher than that of the NC animals. Treatment of the LPS stimulated THP-1 cells with varying concentrations of DAR led to significant reduction in the upregulated production of NFκB protein in a dose-dependent manner. Highest inhibition of NFκB by DAR was found at the concentration of 10 mg/ml (**Figure 11E**).

Overall these *in vitro* results complement well with the *in vivo* study findings and supplement the indication that the DAR is indeed a potent anti-inflammatory and anti-rheumatic herbo-mineral formulation.

## Discussion

Traditional herbal Indian medicine or “Ayurveda” is a widely accepted treatment regime in South Asia for centuries and is gaining popularity in other parts of the world, even as an alternative or additive therapy (Ravishankar and Shukla, 2007; Balkrishna et al., 2019a; Balkrishna et al., 2019b). Disease-modifying anti-rheumatic and anti-inflammatory non-steroidal drugs, either individually or in combination, have been the primary therapy for controlling RA with severe side effects (Kremer, 2001). Compared with synthetic drugs, herbal formulations are considered to be holistic and rather safe, and therefore are considered more suitable for long-term treatments (Karimi et al., 2015). However, these herbal medicines lack scientific evidence in the form of detailed clinical and preclinical studies to prove their efficacy in healing chronic and acute diseases. Traditionally, Amvatari Ras has been prescribed as a medicine for the treatment of RA (Bhaishajya Ratnawali, 18th century A.D.) and has also been tested in some clinical settings (Bhinde et al., 2016). Amvatari Ras and its different components including herbally processed Hg, S, and poly-herbal extracts have been reported to have anti-inflammatory and anti-arthritic properties.

CA-induced inflammation in the rat paws represents a classical model of edema formation and hyperalgesia (Ichitani et al., 1997; Sundy, 2001). In our study, using the CA-stimulated rat RA model, we confirmed that DAR showed anti-inflammatory behavior by reducing inflammatory paw edema proportions.

CAIA Balb/c mice model is a well-studied model for RA disease showing the disease pathogenesis. CAIA mice treated with human equivalent dosages of DAR displayed significant improvement in joint inflammation associated arthritis score, pedal edema, inflammatory tissue lesion, and bone erosion scores. An independent clinical study had shown DAR act as anti-inflammatory and anti-arthritis drug (Singh and Kumar, 2015). However, no pre-clinical study has been performed to understand the mechanism of action of the DAR, an anti-inflammatory and anti-arthritis medicine. The activity of DAR was found similar to the RA standard of care drug, MTX, in reducing RA-associated symptoms. MTX acts on multiple modes, such as by lowering the presence of T-cells at the site of inflammation, by blocking the activity of IL-1β by reducing oxidative stress, and by decreasing bone and cartilage damage (Brody et al., 1993; Bohm, 2004). However, MTX and its metabolites have additional side effects that induce renal and liver failures along with neurotoxicity and gastrointestinal toxicity (Smeland et al., 1996; Shuper et al., 2002; Brugnoletti et al., 2009; Sotoudehmanesh et al., 2010). Xie et al. (2016) had determined the inhibitory concentration 50% (IC_50_) for standard of care medicine, MTX, at 30 μM, equivalent to 13.6 μg/ml tested across several cell lines under *in vitro* conditions. Compared to the MTX, DAR showed an IC_50_ value of 32.19 mg/ml, which was several orders of magnitude higher. The toxicity of MTX and its metabolites has been attributed to its high plasma concentrations either due to high dose or due to its compromised clearance leading to bioaccumulation. Toxicity induced by MTX could also turn fatal, in cases involving elderly patients with decreased or obstructed renal clearance (Shaikh et al., 2018; Arakawa et al., 2019).

DAR is a herbo-mineral formulation consisting of herbally processed mercury, sulfur, multiplant extracts from *T. chebula, T. bellirica, E. officinalis, P. zeylanica, C. mukul*, and *R. communis*. Similar to our study results, treatment of CA-induced inflammation in rat models with *E. officinalis* extracts has exhibited anti-inflammatory properties through a reduction in paw edema (Asmawi et al., 1993; Penolazzi et al., 2008; Sumantran et al., 2008). Likewise, other components of the DAR such as *T. chebula, T. bellirica*, and *P. zeylanica* have also been found to modulate the inflammatory and oxidative damage processes at both the cellular and biochemical levels. The mechanism of modulation is through the inhibition of the production of free radicals, and expression of pro-inflammatory cytokines such as IL-6, TNF-α, LOX-1, COX-2, and NFκB in monocytes (THP-1) and macrophages (RAW 264.7) under *in vitro* conditions, and in CA-stimulated animals (Bag et al., 2013; Yang et al., 2014; Tanaka et al., 2016; Jayesh et al., 2017; Jayesh et al., 2018; Rajalakshmi et al., 2018). Interestingly, Amvatari Ras formulation without Hg has been shown to have lower efficacy in treating RA and associated clinical symptoms as compared to Amvatari Ras containing Hg (Bhinde et al., 2016). However, the absence of Hg in the femoral bone tissue and blood serum in the DAR-treated animals indicated that the Hg content of DAR following treatment did not bioaccumulate or was readily cleared from the target site. International organizations such as World Health Organization (WHO), Food and Agriculture Organization (FAO), United States Environmental Protection Agency (USEPA), European Commission (EC), and United States Food and Drug Administration (USFDA) have defined the safe levels of mercury in the food products such as fish between 0.5 and 5 ppm (FAO/WHO, 1987; WHO, 1990; EC, 2001; US-FDA, 2001; US-EPA, 2004; Burger and Gochfeld, 2005). It is presumed that 50 µg/day intake of organic Hg in an adult would involve a risk of about 0.3% for the development of the symptoms of paraesthesia, whereas an intake of 200 µg/day would involve a risk of about 8% (Kehrig et al., 1998). Hence, our study showed that DAR had a beneficial effect in RA disease as an anti-inflammatory and anti-arthritic formulation without inducing possible side effects from the presence and bioaccumulation of Hg in the joint regions.

The second component of DAR, sulfur, is also well recognized for its anti-inflammatory and anti-RA properties. Sulfur is also an essential component of other herbal formulations used for treating RA (Kumar et al., 2015). It forms the backbone element for several anti-oxidant proteins such as glutathione, nicotinamide adenine dinucleotide dehydrogenase, and the main component of vitamins biotin and thiamine, and amino acids such as methionine, cysteine, homocysteine, and taurine. Treatment of RA patients with different forms of sulfur-containing materials has been observed to have anti-oxidant, anti-inflammatory, and expedited healing processes (Braga et al., 2012; Braga et al., 2013; Santos et al., 2016; Burguera et al., 2017). Though not tested in the present study, being a component of DAR sulfur would have a beneficial effect in reducing RA-associated inflammatory symptoms. In the present study, we found that DAR treatment significantly reduced the liver damage enzymes ALT and AST in the CAIA mice serum. While this is the first reported effect of DAR on liver toxicity, components of DAR, *E. officinalis* and *T. bellirica*, have been individually reported to reduce the severity of chemical-induced hepatic injuries and inhibit the production of ALT and AST enzymes (Tasduq et al., 2005; Kuriakose et al., 2017). Furthermore, we observed that DAR did not induce any additional hepatotoxicity in the CAIA mice.

Using CAIA mice model and LPS stimulated THP-1 cells, we determined the mode of action for DAR’s anti-inflammatory activity in RA disease. Our results showed that DAR behaved as an anti-arthritis herbo-mineral formulation through the five-fold modulation of pro-inflammatory cytokines in a dose-dependent manner. This is an important finding as these pro-inflammation cytokine secretions are directly related to the chronicity of RA disease (Lubberts, and van den Berg, 2003). Pro-inflammatory cytokine TNF-α induces the production of other pro-inflammatory cytokines, such as IL-1 and IL-6, attracting leukocytes from the blood into the inflamed tissue leading to the destruction of the underlying articular cartilage and subchondral bone through the production of proteolytic and metalloproteinase enzymes (Feldmann and Maini, 2001; Sattar et al., 2003). IL-1β and the natural IL-1 receptor antagonist (IL-1ra) are expressed in abundance in the synovial membrane and are involved in cytokine production by synovial mononuclear cells, prostanoid and metalloprotease release by fibroblasts, catabolism and cytokine production by chondrocytes, and bone erosion by osteoclasts (Dayer, 2003). IL-6 cytokine performs pleiotropic functions including effects on the maturation and activation of B and T cells, macrophages, osteoclasts, chondrocytes, and endothelial cells and also has broad impact on hematopoiesis in the bone marrow (Kishimoto, 2005). Treatments with TNF-α, IL-1β, and IL-6 cytokine blockers such as Etanercept (Enbrel), Adalimumab (Humira), and Tocilizumab (Actemra) have shown reduction in symptoms and relief in case of the inflammatory disease. In the present study, the inhibition of pro-inflammatory cytokines especially the observed IL-6 levels under *in vitro* and *in vivo* conditions following DAR treatment could be correlated to a decrease in the pathogenesis of the RA disease in the animal models. RA pathogenesis in the CAIA animals has been reported to be linked to the expression of NFκB (Han et al., 1998). NFκB protein plays a central role as a mediator for stimulating the release of pro-inflammatory cytokines IL-1β and TNF-α, in both innate and adaptive immune cells. Hence, in our study, a fivefold reduction in NFκB expression along with the downstream associated pro-inflammatory cytokines presents the mode of action of DAR in attenuating RA pathogenesis. *T. bellerica* and *E. officinalis* plant extracts have also been reported to modulate apoptosis, inflammation, and oxidative stress by reducing the expression of NFκB protein in different cells and animal models (Penolazzi et al., 2008; Yadav et al., 2017; Tanaka et al., 2018).

During severe inflammation such as RA, the threshold for nociceptor neurons involved in conduction and transmission of signals to fire action potentials is reduced, leading to elevated pain sensitivity or “hyperalgesia” (Kidd and Urban, 2001; Pinho-Ribeiro et al., 2017). This further reduces tissue immune response to damaging stimuli and noxious elements. In the present study, using the Randall-Selitto test and hot plate test, we showed higher pain sensitivity in the CAIA animals following the onset of RA. Treatment of the CAIA animals with DAR helped in the reduction of mechanical and thermal hyperalgesia back to the basal levels similar to those observed using the standard care drug MTX. This indicated the neuroprotective behavior of DAR similar to MTX.

We conclude that DAR is capable of reducing RA-related inflammation and associated clinical symptoms. While synthetic drugs may produce rapid relief from RA-associated edema and pain, their long-term usage and effects on health have been always questionable. Herbal formulations, on the other hand, may have milder efficacious effects in modulating disease-associated symptoms; however, due to their nature-derived origin, and long-term historical use, no adverse effects are expected. In these lines, DAR presents a safer and efficient therapeutic alternative treatment of RA disease.

## Author Contributions

AB provided broad direction for the study, prepared the formulations, generated resources, and gave final approval for the manuscript. SSS conducted the *in vivo* study, analyzed the data, and helped in manuscript writing and reviewing. KhJ, SP, and DJ assisted in animal handling and in conducting *in vivo* studies. RR performed the *in vitro* experiments and reviewed the manuscript. KaJ prepared the histopathological slides. AG supervised the studies and reviewed the manuscript. KB performed data curing and wrote the manuscript and its revisions. AV conceptualized and supervised overall studies, generated resources, and reviewed and finally approved the manuscript.

## Conflict of Interest Statement

The authors declare that the research was conducted in the absence of any commercial or financial relationships that could be construed as a potential conflict of interest.
