# Supplementary figures and images for: Anti-Inflammatory and Anti-Arthritic Efficacies of an Indian Traditional Herbo-Mineral Medicine “Divya Amvatari Ras” in Collagen Antibody-Induced Arthritis (CAIA) Mouse Model Through Modulation of IL-6/IL-1β/TNF-α/NFκB Signaling
Source: Front Pharmacol. 2019 Jul 1;10:659. doi: 10.3389/fphar.2019.00659 (PMC6614787; doi:10.3389/fphar.2019.00659)

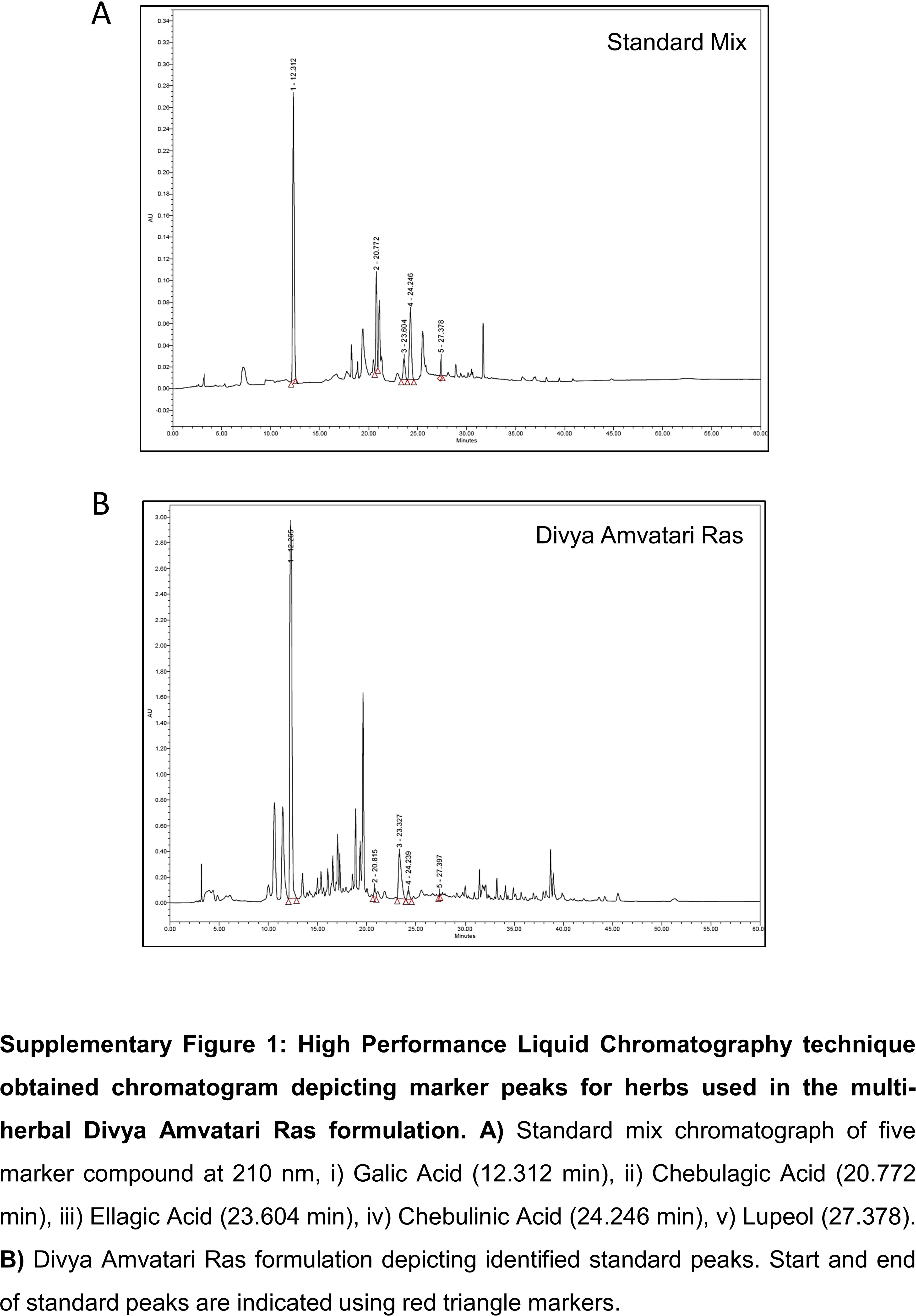

Supplement: Supplementary file 1 [file Image_1.jpeg]

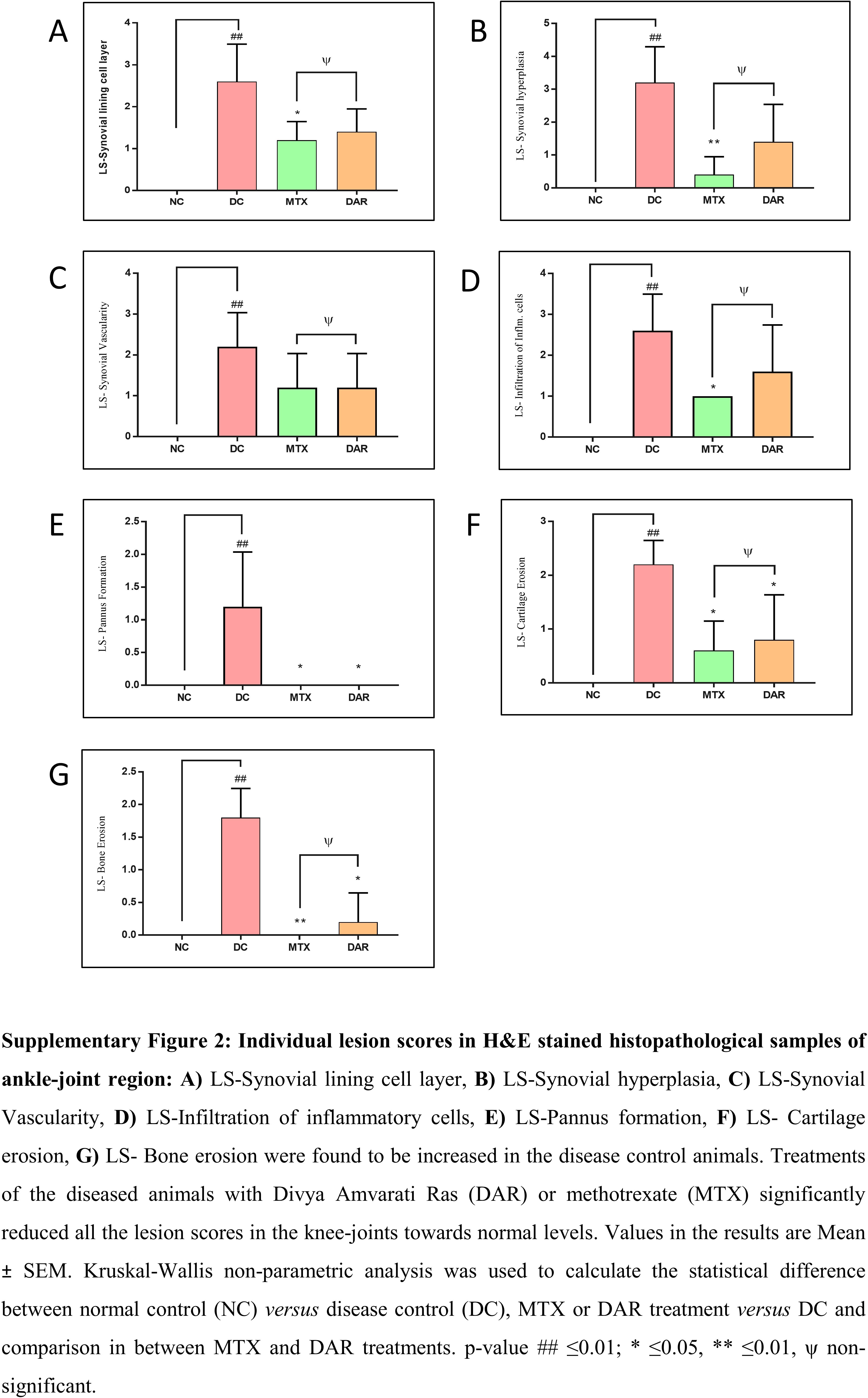

Supplement: Supplementary file 2 [file Image_2.jpeg]

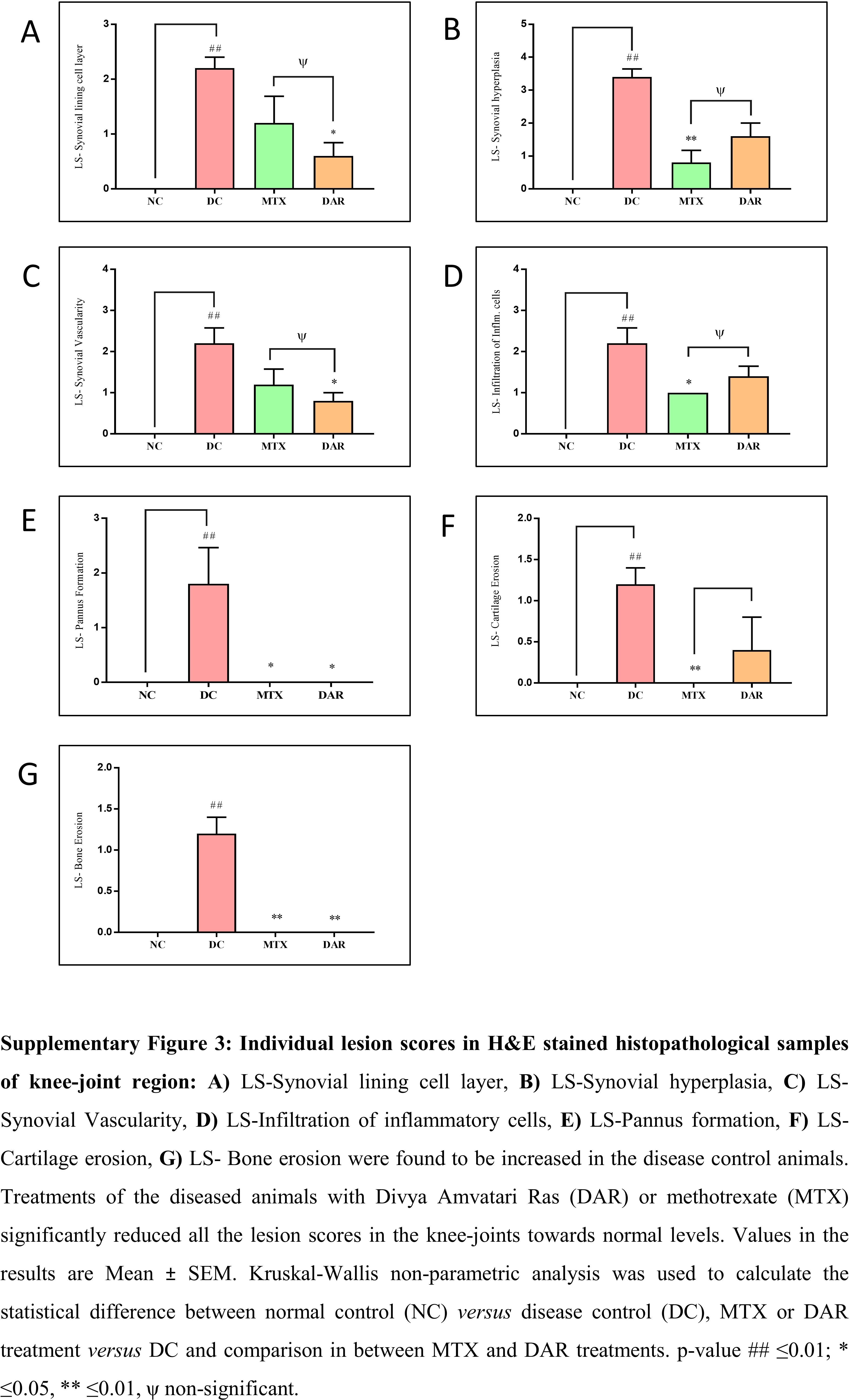

Supplement: Supplementary file 3 [file Image_3.jpeg]
